# MiR-125a enhances self-renewal, lifespan, and migration of murine hematopoietic stem and progenitor cell clones

**DOI:** 10.1038/s41598-019-38503-z

**Published:** 2019-03-18

**Authors:** Edyta Ewa Wojtowicz, Mathilde Johanna Christina Broekhuis, Ellen Weersing, Alexander Dinitzen, Evgenia Verovskaya, Albertina Ausema, Martha Ritsema, Erik Zwart, Gerald de Haan, Leonid V. Bystrykh

**Affiliations:** Laboratory of Ageing Biology and Stem Cells, European Research Institute for the Biology of Ageing, University Medical Centre Groningen, University of Groningen, Antonius Deusinglaan 1, 9700 AV Groningen, The Netherlands

## Abstract

Expansion of hematopoietic stem cells (HSCs) is a ‘holy grail’ of regenerative medicine, as successful stem cell transplantations depend on the number and quality of infused HSCs. Although many attempts have been pursued to either chemically or genetically increase HSC numbers, neither clonal analysis of these expanded cells nor their ability to support mature blood lineages has been demonstrated. Here we show that miR-125a, at the single cell level, can expand murine long-term repopulating HSCs. In addition, miR-125a increases clone longevity, clone size and clonal contribution to hematopoiesis. Unexpectedly, we found that miR-125a expanded HSCs clones were highly homogenously distributed across multiple anatomical sites. Interestingly, these miR-125a overexpressing cells had enhanced mobility and were more frequently detected in the spleen. Our study reveals a novel, cell-intrinsically controlled mechanism by which HSC migration is regulated.

## Introduction

Hematopoietic stem cell (HSC) transplantation constitutes an important treatment modality for multiple hematological disorders, including leukemia. Successful stem cell transplantation largely depends on the number of HSCs that is infused and engrafts. Strategies to improve the efficiency of bone marrow (BM) reconstitution after HSC transplantation have focused on attempts to increase homing of HSCs to the BM or alternatively to expand HSCs *ex vivo* using chemical^1^ or genetic approaches^2^. Although there has been progress in developing HSC expansion protocols^3^, their value for the clinics is still under debate. Whereas under normal conditions HSCs are retained and engraft locally in the BM it is postulated that there may be a maximal capacity of the bone cavity to host HSCs, and expansion beyond such limit may result in HSCs egressing to the circulation resulting in extramedullary hematopoiesis. Interestingly, various inbred strains of mice have different sizes of the HSC pool^4,5^, and increased stem cell pool size in these strains correlate with the efficacy to induce HSC mobilization from bone marrow to blood^4^.

To identify molecular contributors to these genetically regulated qualitative and quantitative HSC-intrinsic differences, we performed genome-wide mRNA^6^ and microRNA^7^ expression studies. The latter analysis revealed an increased expression of the microRNA-99b-let7e-125a cluster in the DBA/2 strain, a strain that displays increased HSC numbers and enhanced mobilization compared to C57BL/6^7^. It appeared that miR-125a largely accounted for the proliferative advantage and increased self-renewal in cells overexpressing this miRNA cluster^7,8^.

To develop alternative strategies to improve hematopoietic reconstitution after transplant, we recently showed that it is feasible to induce functional stem cell activity in progenitors, which are normally devoid of long-term repopulating potential by enforcing expression of miR-125a in committed progenitors. In the current paper we asked whether, at the single cell level, miR-125a overexpression could truly expand murine long-term repopulating HSCs and how this would affect the peripheral blood cell contribution of these cells over time. In addition, we explored whether enforced HSC expansion is associated with ‘saturation’ of the stem cell supporting potential of the BM, and whether saturation leads to stem cell migration. To this end we used a state-of-the-art cellular barcoding method to trace the clonal behavior and blood contribution of expanded HSCs and progenitors and analyze their skeletal allocation. We document for the first time the feasibility of clonal expansion of HSC and progenitors. Mir-125a strongly increased HSC clone number, clone size, clone longevity, and migration, leading to symmetrical distribution of clones throughout the skeleton. Furthermore, these cells showed increased responsiveness to G-CSF *in vitro* and *in vivo* and downregulation of c-Kit expression. We employed a mathematical model, which suggested that an increased self-renewal and slower differentiation rate of HSCs overexpressing miR-125a contribute to their expansion.

## Results

### MiR-125a overexpression increases the number and the size of HSPC clones

**Figure 1:**
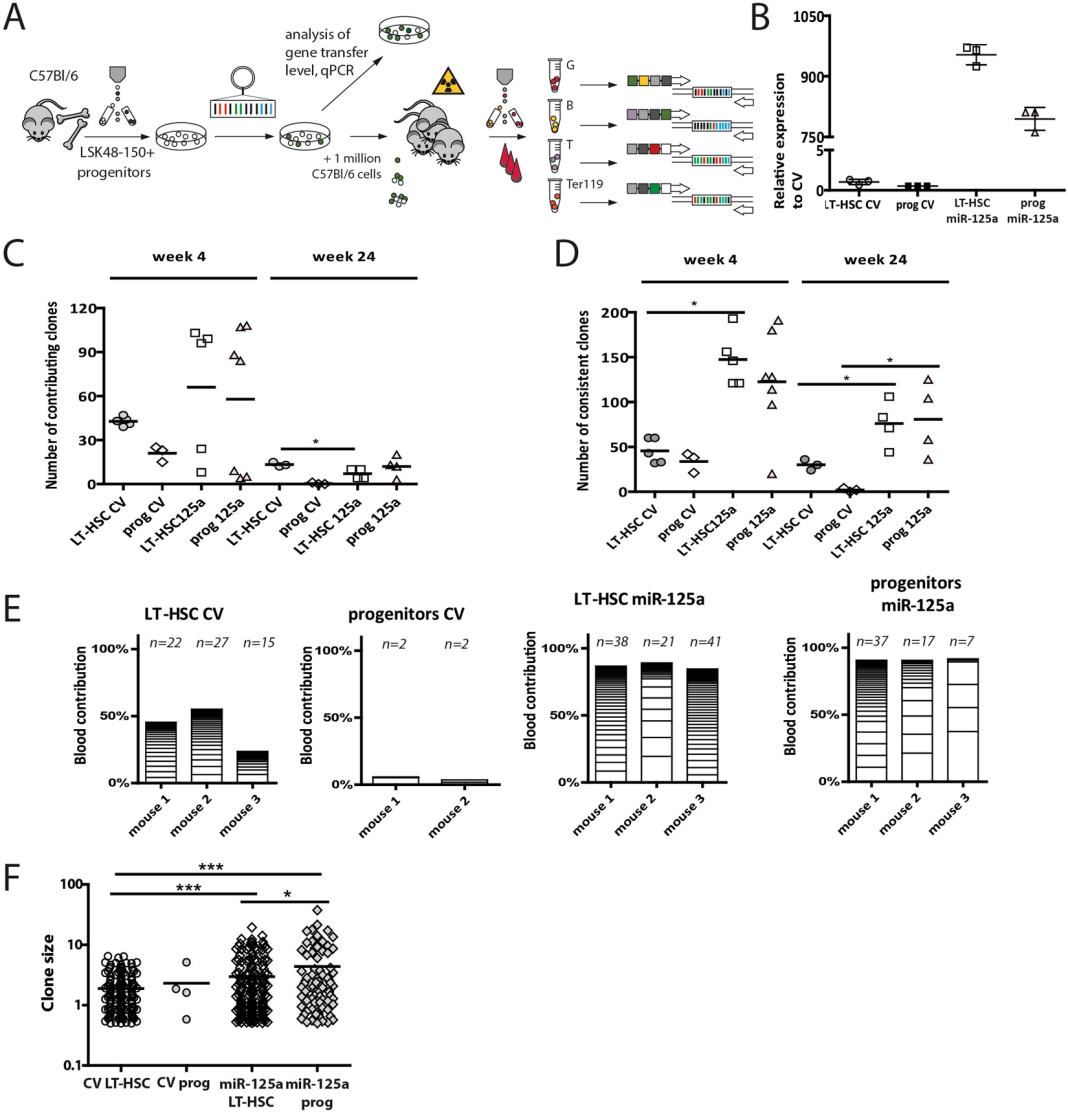
Ectopic miR-125a expression expands LT-HSC pool, endows the self-renewal potential to progenitors and increases clone size. (**A**) Experimental set up. Lin^−^Sca^+^Kit^+^CD150^+^48^−^ (LT-HSCs) were separated from the remaining Lin^−^Sca^+^Kit^+^ cells (progenitors) by FACS, and transduced with barcoded control vector or miR-125a libraries. Transduced cells were transplanted into lethally irradiated C57BL/6 mice. Recipient mice were bled every 4 weeks to FACS-sort Gr-1+, Ter-119+, B220+ and CD3+ cells for barcode analysis. (**B**) qPCR data showing the relative overexpression level of miR-125a to empty vector. Sno202 was used for the normalization. (**C**) Number of clones that contributed >0.5% to myeloid lineage 4 and 24 weeks post transplantation (unpaired student t-test, *p < 0.01). (**D**) Number of consistently contributing clones detected 4 or 24 weeks post transplantation in myeloid lineage (unpaired student t-test, *p < 0.01). Each symbol represents a single mouse, (**E**) Myeloid clone sizes (contributing >0.5% to granulocytes) in blood at 12 weeks post transplantation in control progenitors, miR-125a overexpressing progenitors, control LT-HSC and miR-125a overexpressing LT-HSC Above each column the number of contributing clones (>0.5%) is depicted. (**F**) Summary of clone sizes depicted in panel E (in peripheral blood), miR-125a OE progenitors form larger clones as compared to CV LT-HSC (p < 0.0001, unpaired t-test, two-tailed) and miR-125a OE LT-HSC (p = 0.015, unpaired student t-test). MiR-125a OE LT-HSC form significantly larger clones as compared to CV LT-HSC (p < 0.0001, unpaired t-test, two-tailed).

Counting HSCs and their progeny, as well as clonal analysis of the hematopoietic lineages has been a technical challenge for a long time. Recently, implementation of cellular HSC barcoding has allowed unprecedented insight into clonal behavior of HSCs. Principles and concepts of this method have been described in several recent reviews^9,10^. Here we used a cellular barcoding method to accurately quantify numbers and contribution of stem cells and progenitors to blood lineages to follow the dynamics and longevity of hundreds of individual clones. We isolated LT-HSC (defined as Lin^−^Sca-1^+^cKit^+^CD150^+^CD48^−^ cells^11^) and progenitors (defined as Lin^−^Sca-1^+^cKit^+^ cells, depleted from CD150^+^CD48^−^ cells^12^, (for gating strategy see Supplementary Fig. 1) and transduced these with control or a miR-125a overexpressing barcoded libraries prior to transplantation in two cell doses into lethally irradiated recipients (Fig. 1A). MiR-125a overexpression levels are shown in Fig. 1B, and transplanted cell doses are provided in Supplementary Table 1. We collected blood samples every 4-weeks and FACS-purified granulocytes (SSC^hi^Gr-1^+^), B cells (B220^+^), T cells (CD3ε^+^) and nucleated erythroid cells (Ter-119^+^)^13^ in cohorts of mice transplanted with LT-HSC (n = 5) or progenitors (n = 3) transduced with barcoded control vector (CV), or with LT-HSC (n = 8) or progenitors (n = 7) transduced with barcoded miR-125a vector. We extracted genomic DNA, amplified barcode sequences and subjected samples for sequencing as previously described^14^. Clones were counted in two different ways. We refer to clones as “consistent” if they were reproducibly detected in series of at least 2 consecutive samples. In this case no restrictions were made on their actual contribution to blood production. Using this parameter we counted up to 50 clones in mice transplanted with LT-HSCs or progenitors transduced with CV at week 4 post-transplant, whereas up to 200 clones were detected in mice transplanted with miR-125a OE cells (Fig. 1C). Although after 24 weeks we observed a decrease in the number of clones in all analyzed groups (Fig. 1C, Supplementary Fig. 2A–D), still miR-125a overexpressing cells retained an increased number of stably detected clones compared to control cells. Alternatively, when we focused on clones that contributed to the myeloid lineage more than 0.5% (we refer to these as ‘contributing clones’) the number of clones was smaller for all time points, since the contribution of a considerable number of detected clones was below this threshold (Fig. 1D, Supplementary Fig. 3A–D). We did observe a correlation between the number of transplanted HSC and the number of contributing clones (Fig. 1D), and did detect a significant difference between CV and miR-125a OE cells at 24 weeks post transplantation.

As expected, control progenitors reconstituted lethally irradiated recipients poorly (0–2% and only 0–4 barcodes detected in all analyzed animals), did not contribute to long-term hematopoiesis (Fig. 1E, second panel), and therefore this group was excluded from further analysis. The contribution of control LT-HSC clones to myeloid reconstitution varied from 0.5% up to maximally 6% (Fig. 1E first panel, Supplementary Excel File provides blood contribution of each clone). Our results show that the increased engraftment of miR-125a OE cells coincides with a higher number of clones that significantly contribute more to hematopoiesis, from 0.5% up to 16% to short-lived granulocytes (Fig. 1E, third and fourth panel). These data show an increased number of HSC and progenitor clones that efficiently engraft and differentiate upon miR-125a OE and sustain robust reconstitution upon transplantation (chimerism levels reaching almost 100% already 4 weeks post-transplant, Fig. 1E). Interestingly, miR-125 OE LT-HSCs and progenitors formed significantly larger clones compared to CV LT-HSC (Fig. 1F).

**Figure 2:**
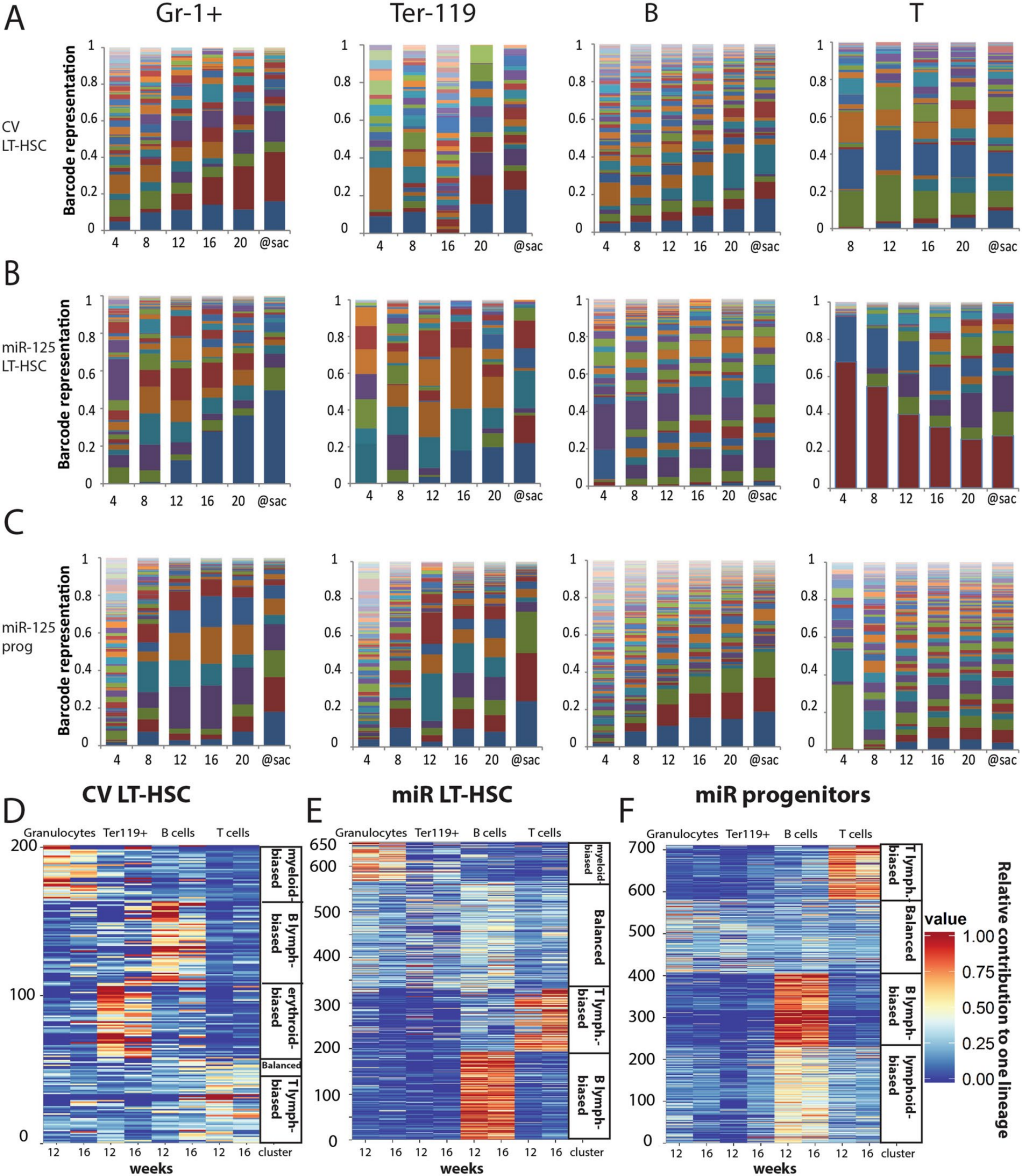
MiR-125a mildly influences lineage choice decision. (**A**) Barcode composition in granulocytes, erythroid cells, B cells and T cells in a mouse transplanted with CV LT-HSC (**B**) A mouse transplanted with miR-125a overexpressing LT-HSC (**C**) A mouse transplanted with progenitors overexpressing miR-125a (**D**) Relative lineage contribution of indicated number of clones detected at 12 and 16 weeks post transplantation in mice transplanted with control LT-HSC, (**E**) miR-125a overexpressing LT-HSC or (**F**) progenitors (n = 3 for each condition).

As miR-125a OE expands HSCs at the clonal level, we asked to what extent each clone contributed to the production of granulocytes, B-cells, T-cells, and erythroid cells (examples of multilineage clonal composition are shown in Fig. 2A–C). Therefore, we compared the lineage contribution of ~200 control LT-HSCs, ~650 miR-125a-expressing LT-HSCs and ~700 miR-125a expressing progenitors (Fig. 2D–F). As has been reported before, granulocytes and lymphocytes are often produced by different subsets of primitive cells^14,15^. Interestingly, here we detected a separate group of clones that preferentially supported the erythroid lineage in normal LT-HSC (Fig. 2D). This primitive cell subset has remained unnoticed in previous studies, most likely because analysis of the erythroid lineage was omitted or performed only once at terminal analysis precluding the kinetic studies^14–16^. We did not detect this group of clones in cells overexpressing miR-125a (Fig. 2E–F). Moreover, in mice transplanted with miR-125a OE progenitors we identified a subgroup of lymphoid biased primitive cells (mainly supporting B-cells and to a smaller extent T cells) that constituted ~1/3 of all detected clones (Fig. 2F). This subgroup was functionally different from the group exclusively supporting B cell production and was absent in all other analyzed groups (Fig. 2F). Unexpectedly, we did not observe any clones preferentially contributing to the myeloid lineage in mice transplanted with miR-125 OE progenitors, which were expected based on miR-125a induced myeloid skewing and myeloproliferation^7,8,17–19^. However, in miR-125a OE LT-HSC we did detect ~10% of myeloid biased clones and an increased fraction (over 1/3 of all analyzed clones) of balanced HSCs, supporting all mature blood lineages (Fig. 2E).

In the majority of analyzed mice we observed that different clones supported different mature blood lineages (Supplementary Fig. 4A). However, there was one exception: in mice transplanted with miR-125a OE LT-HSC and progenitor cells we observed a higher overlap between barcodes detected in erythroid and myeloid lineages as compared to control LT-HSC (Supplementary Fig. 4A–C). Therefore, expanded LT-HSCs and progenitors reveal a mild alteration in the lineage commitment.

### MiR-125a overexpression extends clonal life span upon serial transplantation

**Figure 3:**
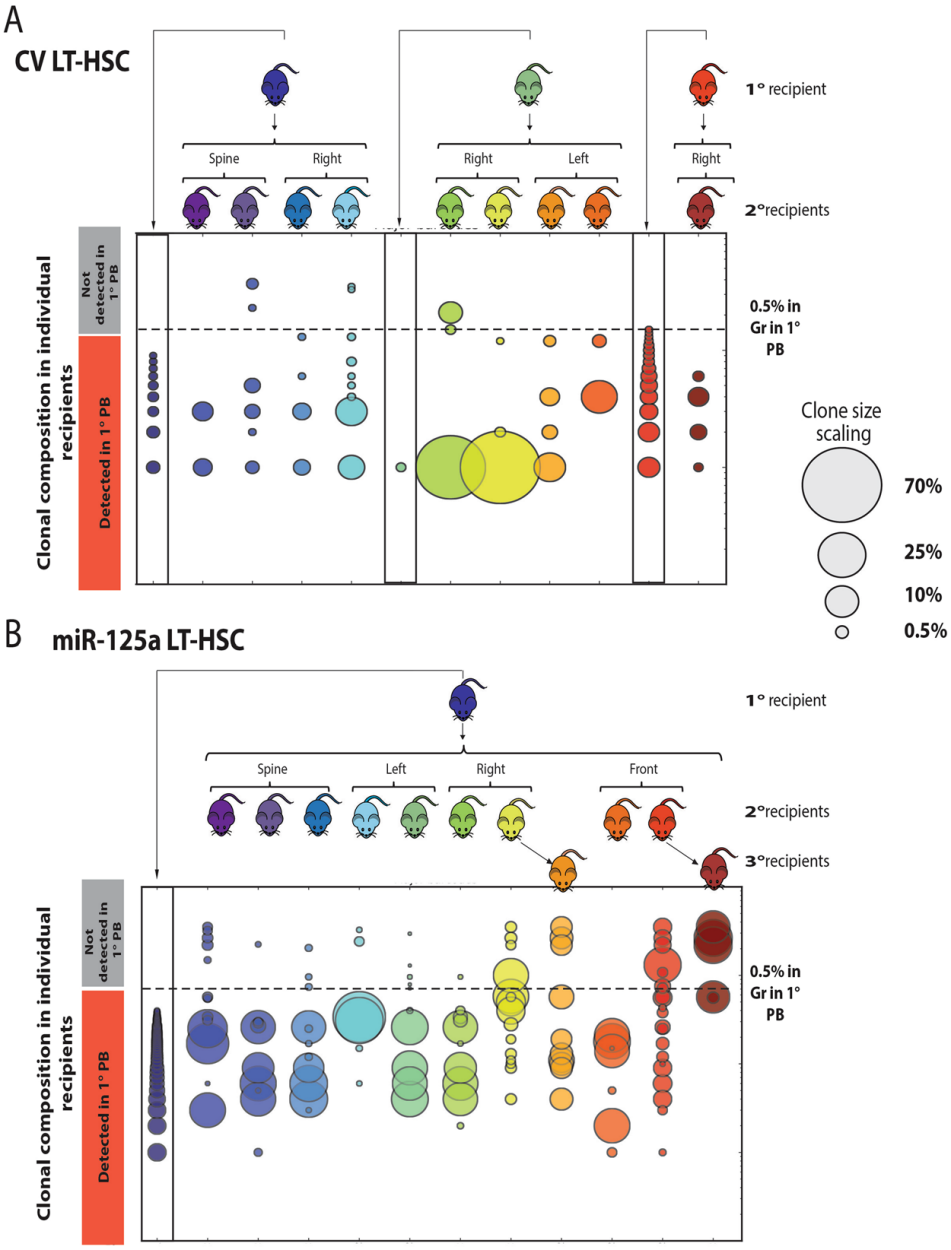
MiR-125a extends clonal lifespan, while serial transplantation ‘activates’ dormant clones (**A**) Serial transplantations of control LT-HSC or (**B**) miR-125a overexpressing LT-HSC. Barcoded bone marrow cells were isolated from multiple skeletal regions of primary recipients, and serially transplanted (origin of cells depicted over mouse pictogram). Clonal make-up in secondary (and tertiary) mice was compared with clonal make-up of the primary recipient that served as bone marrow donor. The color code indicates which clones were originating from which mouse. Size of the clone is indicated by the size of the circle (to estimate the relative contribution to granulocytes, the barcode representation in granulocytes was divided by total barcode representation (granulocytes+ Ter-119+ T cells+ B cells). Many clones that were detected in the peripheral blood of secondary recipients were also present in the blood of the primary recipient (detected in 1° PB). However, some secondary clones were only present in the bone marrow of the primary recipient (indicated by ‘not detected in 1° PB′). Only clones that contributed at least 0.5% to Gr-1+ at week 16 are shown for the primary recipient).

To assess whether miR-125a-induced expansion affects HSC clonal self-renewal or clonal lifespan, we collected cells isolated from various skeletal locations (spine, right or left femur, front legs and sternum or skull) from individual primary recipients and transplanted 5 million cells into lethally irradiated secondary and tertiary recipients (Fig. 3A,B, Supplementary Fig. 5A,B). We repeatedly bled mice and analyzed the clonal composition in Gr-1^+^ SSC^hi^ cells 10 or 12 weeks after 2^nd^ and 3^rd^ transplantation. In these figures (Fig. 3A,B) each detected barcode is depicted as a circle, where the size of the circle indicates clone size (the scaling of clone size is shown in the figure, the colors of the circle indicate the skeletal origin of BM cells transplanted into recipient mouse). We analyzed the clones present in peripheral blood (depicted as detected in 1° PB) and bone marrow (depicted as not detected in 1° PB) in the primary recipient. Not all clones detected in the BM were present in PB. Next, we compared the clones detected in PB of secondary and tertiary recipients with those found in PB and BM of the primary recipients. We have observed that some of clones found only in the BM of the primary recipient contributed to PB hematopoiesis in serially transplanted recipients (detected clones in primary recipients are shown within black rectangles - the 1^st^, 6^th^ or 11^th^ column of circles in Fig. 3A or the 1^st^ column in Fig. 3B). We ranked the detected barcodes in the PB of each primary recipient from the highest to lowest contributing clones (counting only clones contributing >0.5% to granulocytes Fig. 3A,B, Supplementary Fig. 5A,B). Same barcodes detected in blood of primary and serially transplanted recipients are plotted in the same row. In 7 out of 9 secondary recipients transplanted with CV LT-HSC we detected clones that were not active in the primary recipient, but significantly contributed to hematopoiesis upon serial transplantation (Fig. 3A, Supplementary Fig. 5A,B). In secondary recipients transplanted with CV LT-HSC we observed that the number of clones contributing to the myeloid lineage was low (on average 4 clones) with low contribution to hematopoiesis, therefore precluding a 3^rd^ round of transplantation. We detected a similar pattern of ‘reactivated’ clones in mice serially transplanted with miR-125a OE LT-HSC (Fig. 3B, Supplementary Figs 5C,D and 6A,B for two additional serial transplantation experiments). However, miR-125a expanded cells survived and repopulated lethally irradiated recipients more robustly compared to CV LT-HSC, since secondary and tertiary recipients were highly chimeric (80–90% chimerism in SSC^hi^Gr-1^+^ cells) and showed an increased number of contributing clones, compared to control (Fig. 3B, Supplementary Figs 5A,B, 6A,B).

To summarize, CV LT-HSC clones that were dominant in primary recipients displayed only restricted contribution to hematopoiesis in serial transplantations. In contrast, miR-125a expanded cells showed significantly extended clonal longevity and function, resulting in more clones with higher contribution to hematopoiesis in serial transplantations.

### Multiple viral integrations and dose effect

We asked whether an increased dose of miR-125a expression, induced by multiple miR-125a copies in the same cell, would be associated with enhanced stem cell activity and whether these cells would be positively selected for in the course of serial transplantations. The odds of a cell being infected with multiple viruses increases proportionally to the transduction efficiency (Supplementary Fig. 7A). When barcoded vectors are used, such events can be recognized by an unusually high degree of correlation between multiple barcodes.

To monitor clones and describe their competitive behavior over time we analyzed their blood cell contribution by ranking barcodes in primary and secondary recipients according to clone size in PB and BM. Strikingly, in CV LT-HSC and CV progenitors we detected fewer clones with multiple vector copy number per cell than expected by chance (Supplementary Fig. 7A,D,E). This suggests that multiple integrations of the vector in the genome may be detrimental to cells. This observation is in contrast to the common believe that multiple hits in the genome are prone to positive selection^20,21^. In contrast, miR-125a expanded stem and progenitor cells followed frequencies of single and multiple vector copy number integrations as expected by chance, implying no selective pressure on the vector copy number counted per cell in primary recipients (Supplementary Fig. 7B,C). However, we did observe positive selection for multiple hit clones overexpressing miR-125a in the 2^nd^ round of transplantation (Supplementary Fig. 7F,G). This suggests that better engraftment, extended lifespan and increased self-renewal is indeed miR-125a dose-dependent.

### MiR-125a induced expansion of HSCs results in homogenous distribution of clones throughout the skeleton

**Figure 4:**
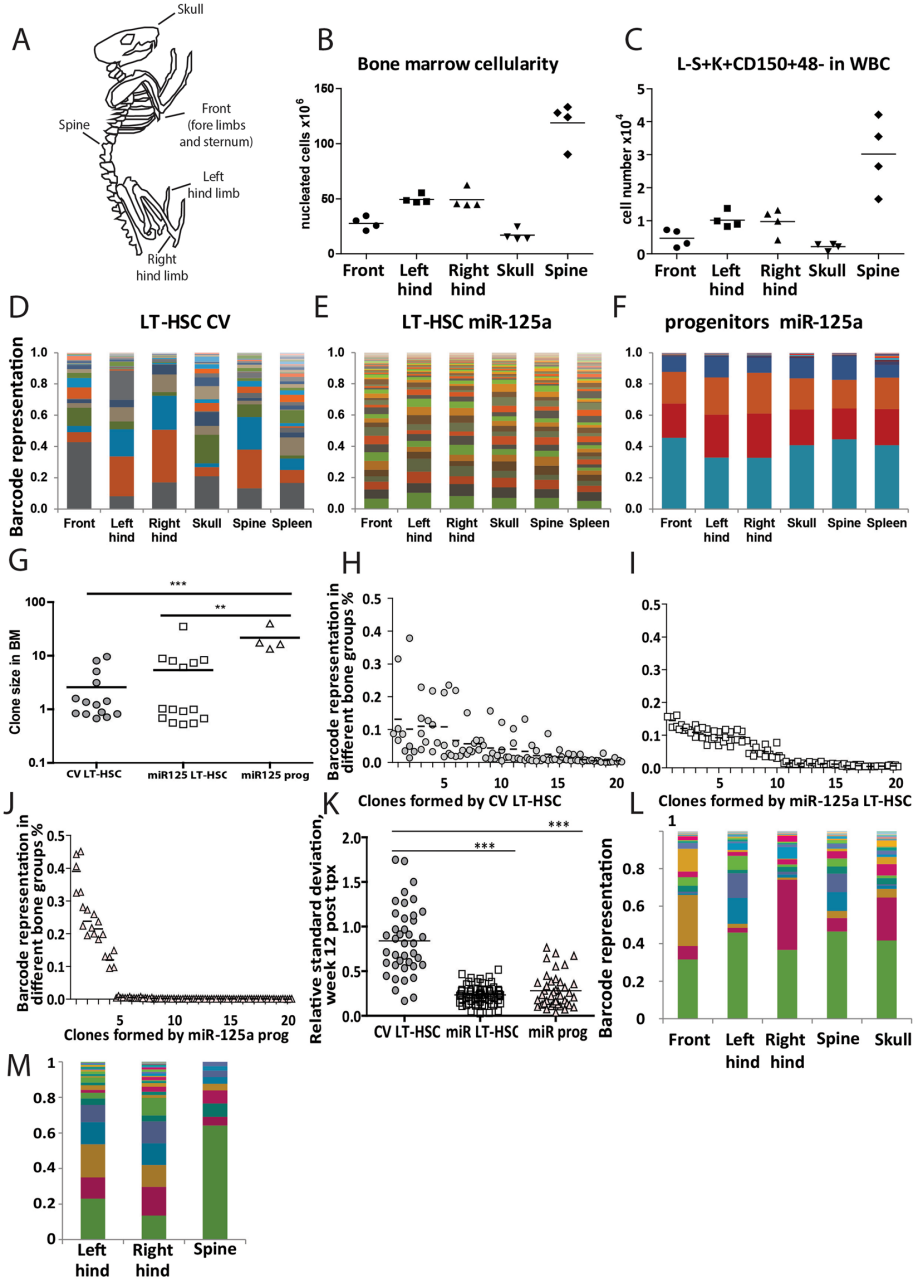
MiR-125a overexpression redistributes clones across the skeleton. (**A**) Schematic representation of the 5 bone groups that were analyzed. (**B**) Bone marrow cellularity (expressed as a total number of nucleated cells) and (**C**) Total number of LT-HSC (Lin-Sca+ Kit+ CD150+ 48-) isolated from various skeletal locations (**D**). Skeletal distribution of barcoded clones in control LT-HSC, (**E**) miR-125a overexpressing LT-HSC, or (**F**) Progenitors. (**G**) Comparison of the clone sizes formed in the bone marrow (of mice depicted in **D**–**F**). Progenitors OE miR-125a form fewer, larger clones as compared to CV LT-HSC (p < 0.0001, unpaired Student t-test, two-tailed) or miR-125a OE LT-HSC (p = 0.048, unpaired Student t-test, two-tailed). For the 20 most abundant clones we calculated their relative abundance in the skeletal site where they were found 12 weeks post transplantation in mice transplanted with control LT-HSC (**H**), with miR-125a overexpressing LT-HSC (**I**), and with miR-125a overexpressing progenitors (**J**). Similar patterns were observed in other analyzed mice (data not shown). (**K**) Relative standard deviations for the distribution of clones found in mice transplanted with CV LT-HSC (n = 2), miR-125a LT-HSC (n = 3), miR-125a progenitors (n = 2) at 12 weeks post transplantation. Horizontal lines indicate the mean value. The difference between the groups was assessed by two-tailed Mann-Whitney test (***P < 0.0001). (**L**,**M**) The skeletal distribution of miR-125a OE clones when transplanted at lower dose (cell dose in Suppl. Table 1), depicted are two individual mice.

Since miR-125a significantly expands the number of clones that support hematopoiesis, we wished to address whether the expanded HSC pool would exceed the capacity of the BM to host them. To this end cells were obtained from front limbs and sternum, the left hind limb, the right hind limb, the skull, and from the spine (Fig. 4A). Both the total yield of nucleated cells (Fig. 4B) and LT-HSCs (Fig. 4C) was distinct for each site. Particularly spine turned out to be a wealthy, and generally overlooked, source of LT-HSCs. Mice transplanted with CV LT-HSC, miR-125a OE LT-HSC or progenitors were sacrificed at 12 or 24 weeks post transplantation. We isolated cells from various locations and sorted Lin^−^c-kit^+^ cells from those to assess barcode distributions.

As we and others have showed before, the distribution of barcoded clones derived from control HSCs in these anatomical sites was highly asymmetrical (Fig. 4D, Supplementary Fig. 8A for more examples)^22,23^. Clones that were dominant and constituted ~20% of all clones at one site, were often barely detectable at other sites. This suggests that upon transplantation, HSC seed a local BM niche and not readily egress to the circulation^22^.

In contrast, the clonal makeup in mice transplanted with barcoded miR-125a expanded LT-HSCs or progenitors was highly symmetrical (Fig. 4E,F and Supplementary Fig. 8B,C). As shown before in the peripheral blood (Fig. 1F), we observed that miR-125a overexpression increased BM clone size (Fig. 4G). In total we evaluated 7 control and 10 miR-125a transplanted mice, and consistently, in each mouse transplanted with a high number of miR-125a OE cells, primitive cells were symmetrically distributed across all tested locations of the skeleton (exp. 2, Suppl. Table 1) and in the spleen. For any individual control clone, the contribution to the local BM compartment varied widely from 1 to 37% (Fig. 4H, Supplementary Fig. 8D). In contrast, clones formed by LT-HSC or progenitors expanded by miR-125a showed a very narrow size range across all skeletal locations, despite their different size and capacity to host HSC (Fig. 4I,J, Supplementary Fig. 8E,F). These data suggest that miR-125a expanded HSCs saturate the available ‘space’ and colonize other sites including the spleen. In agreement, the barcode clonal make up in the spleen was similar to that found in other analyzed skeletal locations (Fig. 4E,F).

To quantitatively assess the clonal asymmetry we calculated the relative standard deviation (RSD) for the average clone size in different bones (for details see^22^). The mean RSD for CV LT-HSC clones was 0.84, whereas for miR-125a expanded LT-HSC and progenitors the RSD was 0.22 and 0.24, respectively (Fig. 4K). To test whether the asymmetrical skeletal distributions in control mice would equalize in time, we analyzed an additional 3 mice/condition at 24 weeks post transplantation. Again, however, we found a diverse skeletal distribution for CV LT-HSC and a symmetrical distribution for miR-125a OE cells (Supplementary Fig. 8G).

We hypothesized that the miR-125a expanded HSC pool resulted in saturation of BM niches. To test this we transplanted lower numbers of expanded HSC, which would be expected to result in asymmetrical clonal distribution. (Suppl. Table 1, exp.1-cell doses). Indeed, the skeletal distribution of miR-125a expanded HSC clones 24 weeks post transplantation now was highly asymmetrical and had a similar skeletal distribution compared to non-expanded control cells (Fig. 4L,M). These data support our hypothesis that indeed excessive HSC expansion saturates BM niches.

**Figure 5:**
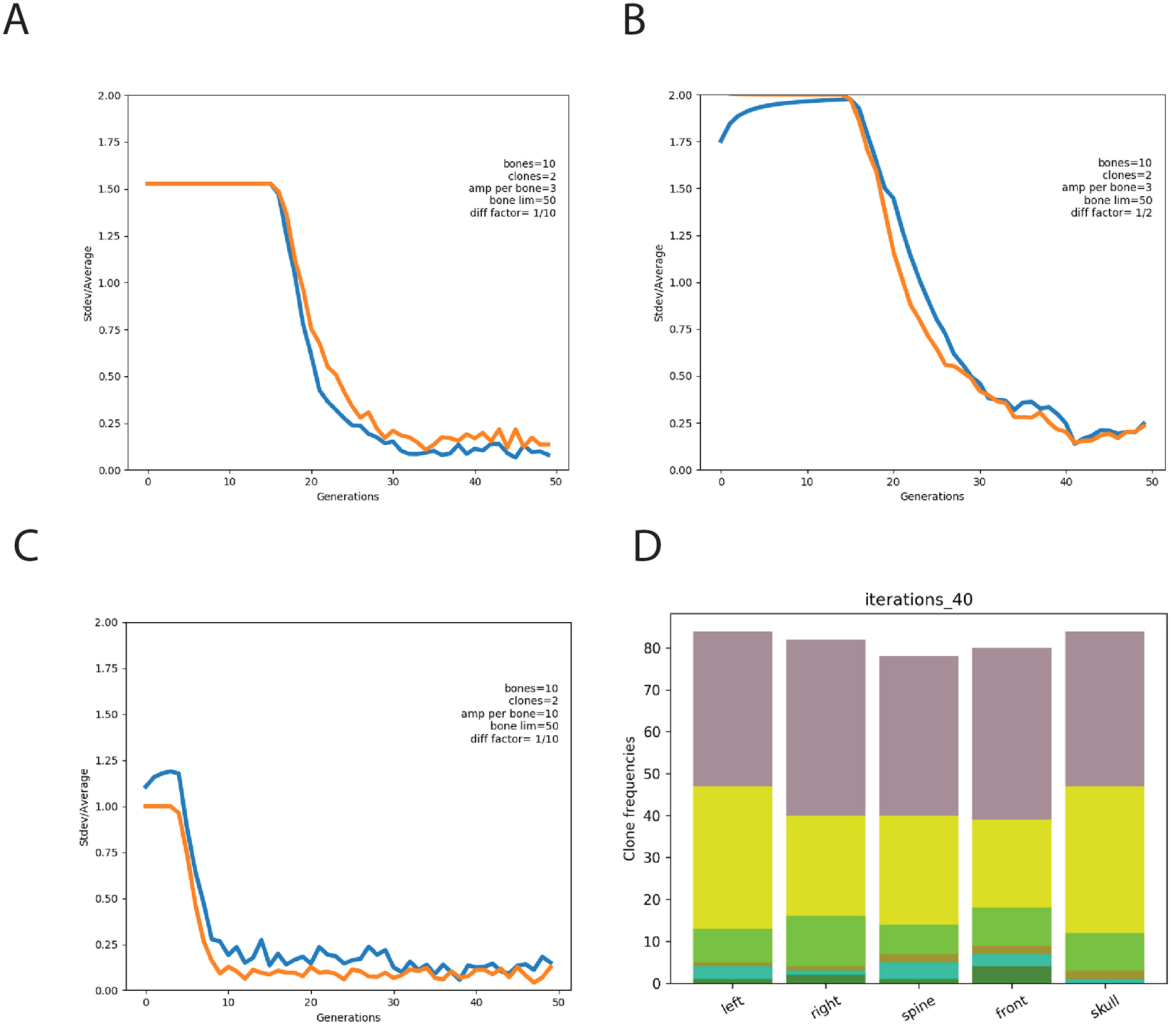
Mathematical modelling of HSC clone equilibration as a function of the size of bone marrow niche, self-renewal and differentiation potential Panels A, B, and C show dynamics of the equilibration of two clones (orange and blue lines) across 10 bones. (**A**) Three major phases of clone dynamics are indicated. Shortly after transplantation clones are seeded at random in 10 bones; a latent phase with a high degree of asymmetry. Once niches become saturated (arbitrary at 50 cells per bone), clonal equilibration begins and cells leave the bone and enter the circulation, resulting a stationary phase with low asymmetry. Note, that by changing the differentiation factor (**A**) versus (**B**), equilibration is affected. If the self-renewal rate is altered (**A**) versus (**C**), the latent phase becomes shorter and equilibration is more rapidly achieved. We refer to a state of equilibrium when clones become equal in size and no further decline of StDev/Average occurs. More details are given in the supplementary Powerpoint presentation. (**D**) Snapshot of the clonal dynamics at equilibrium; the complete dynamic gif file can be found in the supplementary presentation.

To explore the contribution of stem cell dose and stem cell clone size to BM niche saturation (measured by the extent of clonal symmetry) we developed a mathematical model of clonal engraftment and clonal distribution (Supplementary Power Point presentation). We defined the number of HSCs (cells) and skeletal locations available for hosting HSCs (bins). The model allows HSCs to locally expand and if their number exceeds the hosting capacity of the BM compartment, cells are forced to enter the blood and migrate to other locations, or differentiate (the model controls the rate of differentiation). The model repeats this process n-times and plots the resulting skeletal distribution of HSC clones by depicting the relative standard deviation of clonal distribution across the various bone marrow sites. As expected, a higher dose of transplanted cells shortened the phase of initial local, clonal expansion (latent phase) and stimulated rapid clonal equilibration between distinct skeletal locations (Fig. 5A,B). If HSCs that leave the niche would mostly differentiate, clonal disequilibrium could be maintained practically endlessly (Fig. 5C). However, if only a small fraction of migrating cells (in our simulation 1/30^th^ of them) would differentiate, after 30 generations we would observe full skeletal equilibration (Fig. 5D, Suppl. Power Point presentation). As in our experimental studies we observed a faster equilibration across the skeleton upon miR-125a expression (Fig. 4E,F) our model predicts that this results likely from enhanced self-renewal divisions and/or lower differentiation rate.

### MirR-125a expanded HSC and progenitors are migrating in the peripheral blood at steady state and are mobilized in response to G-CSF

**Figure 6:**
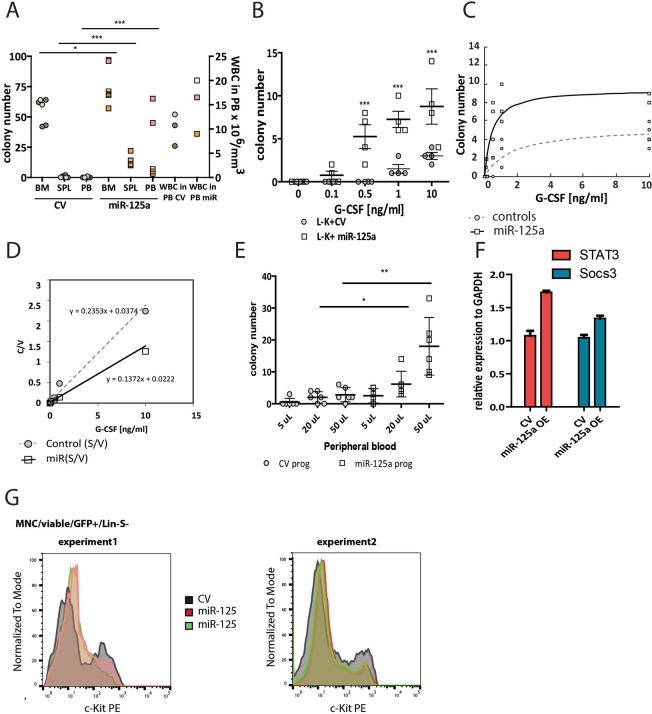
MiR-125a OE induces the presence of CFU-GM in the peripheral blood and enhances their responsiveness to G-CSF (**A**) CFU-GM colonies grown from cells isolated from bone marrow, spleen and peripheral blood. Shown are 3 mice/condition, different colors represent different mice, circles represent mice transplanted with control cells while squares reflect mice transplanted with miR-125 OE cells. The two last data points show the number of white blood cells measured in the peripheral blood at the moment of cell isolation (note right Y-axis), Statistical significant differences were observed between CV and miR-125a OE (BM p = 0.0271, SPL p < 0.0001, PB p < 0.0001 Mann-Whitney non-parametric test). (**B**) The response of Lin-c-Kit+ GFP+ progenitors to increasing concentration of G-CSF *in vitro*, measured as the number of formed CFU-GM colonies. Circles represent control, squares miR-125a overexpressing cells. (**C**) Michaelis-Menten curve for control or miR-125a overexpressing cells, generated using the on-line script: http://ic50.tk/kmvmax.html, (**D**) Hanes – Woolf transformation of Michaelis-Menten equation for control and miR-125a OE cells. (**E**) The number of CFU-GM in increasing volumes of peripheral blood isolated from mice transplanted with control or miR-125a OE cells after G-CSF administration. (**F**) qPCR analysis of STAT3 and Socs3 expression in FACS purified Lin-cKit + eGFP + cells from mice transplanted with CV or miR-125a OE cells. (**G**) c-Kit expression levels in Lin-Sca-Kit + cells isolated from animals transplanted with CV or miR-125a OE cells.

During steady state hematopoiesis, no or very few hematopoietic progenitors can be cultured from the PB. To assess whether miR-125a induced HSC expansion causes BM saturation and results in spontaneous progenitor cell mobilization, we evaluated the number of clonogenic progenitor cells in PB from control and miR-125a transplanted mice. Indeed, the frequency of cells with clonogenic potential was ~10-fold higher in the PB of mice transplanted with miR-125a OE cells compared to controls (Fig. 6A). In addition, we quantified the number of progenitors with clonogenic potential in the spleen to measure extramedullary hematopoiesis, and detected a strong increase in mice transplanted with miR-125a expanded cells (Fig. 6A). There was a moderate difference in the number of colony forming progenitors in the BM of miR-125a or control mice (Fig. 6A), indicating that the progenitor pool in the BM remained largely unaffected by miR-125a OE. To further evaluate whether cells with long-term repopulating ability were also present in the peripheral blood, we isolated Lin-GFP+ cells from primary transplanted recipients and injected these into lethally irradiated secondary recipients. Within tested cell dosage (Supplementary Table 2) we did not observe engraftment. However, mice transplanted with cells isolated from the spleen of miR125a-OE mice fully reconstituted recipients. (Supplementary Table 2). This suggests that miR-125a leads to the migration of stem and progenitor cells from the bone marrow, and accumulation of LT-HSCs in the spleen.

The equal skeletal distribution of miR-125a expanded clones was very similar to patterns we observed previously upon G-CSF administration and is very different from steady-state where clones are asymmetrically distributed in the skeleton^22,23^. As both exogenous G-CSF administration and endogenous miR-125a OE result in skeletal equilibration of clones, we asked whether miR-125a OE would render cells more responsive to exogenously administered G-CSF. To this end hematopoietic progenitors (L^−^K^+^eGFP^+^) were isolated from BM of mice transplanted with control or miR-125a OE LT-HSCs, and subsequently cultured in increasing concentrations of G-CSF (Fig. 6B). Indeed, miR-125a OE led to a strong G-CSF induced increase of their clonogenic potential. In low G-CSF concentration we observed CFU-GM colonies derived from L^−^K^+^eGFP^+^ expressing miR-125a, but not in control cells. Analysis of the dose sensitivity (K_M_) and the maximal response (V_MAX_) of control and miR-125a OE LT-HSC to G-CSF revealed similar sensitivity of both cell types (mice transplanted with CV LT-HSC K_M_ = 1.59 ± 0.69, or miR-125a OE LT-HSC K_M_ = 0.43 ± 0.21). However, the maximal response was higher for miR-125a OE LT-HSC and significantly different from CV LT-HSC (CV V_MAX_ = 5.31 ± 0.77, miR-125a V_MAX_ = 9.48 ± 1.27, Fig. 6C,D).

Encouraged by the *in vitro* data we next evaluated the responsiveness of miR-125a expressing cells to G-CSF *in vivo*, by injecting mice transplanted with control or miR-125a expanded cells with a suboptimal dose of this growth factor. We initiated CFU-GM assays using PB cells isolated 3 days after administration of a single dose of G-CSF, and observed a significantly increased number of colonies formed by cells OE miR-125a compared to control cells (Fig. 6E). Informed by our own and reports from others^12,24^ we screened for miR-125a targets that have been reported to be involved in G-CSF signal transduction and/or cell mobility. We evaluated the expression levels of Socs3, a negative regulator of G-CSF signaling, and a direct target of Stat3 (which is a known target of miR-125a^24^) but we did not observe any difference at the expression level between CV and miR-125a (Fig. 6F). C-Kit, another predicted miR-125a target, has been reported as a key receptor regulating the interaction niche-HSC^25,26^ We observed decreased protein expression upon miR-125a OE (Fig. 6G). Therefore, we postulate that enhanced migration of HSCs upon miR-125 OE is due to downregulation of c-Kit expression, rather than sensitization to G-CSF.

## Discussion

In the current paper we assessed whether, at the single cell level, miR-125a overexpression can truly expand murine long-term repopulating HSCs. We used a state-of-the-art cellular barcoding approach to detect that indeed, miR-125a overexpression causes an increased number of HSC clones that contribute to multilineage engraftment after transplant. In addition, miR-125a substantially increases clone longevity, clone size and clonal contribution to hematopoiesis in long-term serial transplantation experiments. Unexpectedly, we identified cells with a clonogenic potential in the peripheral blood in miR-125a transplanted mice during steady state hematopoiesis. This strongly indicated that miR-125a causes enhanced HSC trafficking from the BM to the circulation. Indeed, when we explored how uniquely barcoded HSCs were distributed across many skeletal sites, we found that, in contrast to wild type HSCs, miR-125a expanded HSCs were highly homogenously distributed. Interestingly, these cells were more sensitive to G-CSF, *in vitro* and *in vivo*, possibly contributing to observed increased cell mobility through downregulation of c-Kit expression. To the best of our knowledge, our data are the first to document the feasibility of clonal HSCs expansion, and of increasing clonal HSC lifespan. Our study reveals also a novel, cell-intrinsically controlled mechanism by which HSC mobilization is regulated.

There are several potential mechanisms that may explain why miR-125a overexpression causes HSC to migrate. First, as miR-125a overexpression results in a significant expansion of the total stem cell pool size, it is possible that the number of niches that is available to support stem cells becomes limiting, and excessive stem cells are forced to migrate to secondary hematopoietic organs. Niche saturation may become even more prominent as we have shown recently that miR-125a overexpression in progenitors induces stem cell potential in these committed cells^12^. Although our molecular and physiological understanding of the HSC niche remains very incomplete, it is interesting to note that in clinical conditions in which the restricted BM niche fails to support stem cells, most notably in osteopetrosis, HSCs leave marrow and seed extramedullary sites^27^.

A second, not mutually exclusive, mechanism why miR-125a overexpression may cause spontaneous HSC mobilization is that it increases the responsiveness of cells to mobilization-inducing cytokines, most notable G-CSF. It is well-known that after G-CSF administration, a selected subset of primitive cells is preferentially mobilized from the BM to peripheral blood^28,29^. It is also interesting to note that genetically regulated differences in miR-125a expression correlate with stem cell pool size and G-CSF induced cell mobilization in DBA/2 and C57BL/6 mouse strains^7^. It is unknown how miR-125a expression is controlled in HSCs, but as the miR-125a *locus* is highly conserved between mice and human, and as we have recently shown that the function of miR-125a is indeed conserved between these two species^12^, we speculate that miR125a levels associate with stem cell expansion and mobilization in humans as well.

It is likely that miR-125a induced expansion and mobilization, which we report in the current paper, is caused by (an interplay of) multiple target genes including c-Kit.

## Materials and Methods

### Mice

C57/BL/6 (B6) mice were purchased from Harlan, C57BL/6SJL were bred in the Central Animal Facility of the University Medical Center Groningen. All animal experiments were approved and performed according to guidelines of the University of Groningen Animal Care Committee.

### Purification and transduction of BM cells

LSK48–150+, LSK depleted from CD48–150+ cell sorting and transduction were performed as previously described^12^. Briefly BM cells from bones of hind legs, spines and sterna of naïve, female B6 mice were crushed and stained with antibodies against Sca-1, c-Kit, CD48, CD150, CD3ε, Gr1, Ter119 and B220. Cells were sorted using MoFlo XDP and MoFlo Astrios (Beckman Coulter) cell sorters and prestimulated in StemSpan medium (STEMCELL Technologies) supplemented with 300 ng/ml stem cell factor (SCF), 1 ng/ml Flt3 ligand (Amgen), and 20 ng/ml IL-11 (R&R Systems) for 24 h. Cells were transduced with viral supernatant containing barcoded MIEV or 633-miR125a vectors in RetroNectin-covered plates (Takara Bio Inc.) in the presence of 2 µg/ml polybrene (Sigma-Aldrich).

### Barcoded libraries

MIEV library was constructed as previously described (Gerrits *et al*., 2010). To obtain 633-miR125a library the vector containing miR-125 was cut with PacI and SalI, synthesized oligos (IDT) were hybridized, ligated into the vector and used for bacteria (DH5α) transformation. Bacterial colonies were grown in 2 ml culture, 4 colonies per tube, then mini-preps were performed, isolated DNA has been stored at-80. All preps were tested by PCR for the barcode region, as well as restriction analysis was done to discriminate barcoded and unbarcoded vectors. Note the current barcode version had a structure: (PacI)-GAATGGNNACNNAGNN**CCATGG**NNCANNCGNNTCTGGCG-(SalI). This included the NcoI restriction site within the barcode sequence (bold). In total we obtained 434 clones that were mixed in equal concentrations and used for transductions. Both vectors used for libraries construction are essentially the same MLV type of the retroviral vector with nearly identical LTR, psi regions and other sites essential for the integration of the virus and expression. The libraries are comparable in size, which allows direct comparative analysis.

### Bone marrow cell transplantation

20–24 h after the first transduction cells were transplanted into lethally irradiated recipients (9 Gy) in the presence of 1 million radioprotective cells from B6 mouse. Efficiency of gene transfer, measured by flow cytometry (LSR-II, BD) is given in Supplementary Table 1. Donors were 4 months old and recipient animals were 2–4 months old. Donor and GFP chimerism were determined in blood every 4 weeks. For secondary transplantations 5 million whole BM cells were transplanted into lethally irradiated recipients. A fraction of mice presented in Fig. 3A (blue and green donor mice) were previously used^22^.

### Q-PCR

MiR-125a overexpression level was measured as previously described^18^. For Socs3 and Stat3 expression we used previously published primers^12,24^.

### G-CSF administration

To test the mobilization rate in control and miR-125a overexpressing cells, we performed one injection of PEGylated G-CSF intraperitoneally (25 µg/mouse dissolved in PBS) 3 days prior the sacrifice. To confirm the efficiency of the mobilization we also injected 1 naive B6 mouse and scored CFUs in the blood sample.

### Colony unit forming cell assay (CFU-GM)

To test the clonogenic potential of control and miR-125a overexpressing cells non-treated with G-CSF we plated 100 000 cells isolated from the spleen or bone marrow or 1 capillary (50 µL) of peripheral blood and scored colonies at day 7.

To analyze the sensitivity of control or miR-125a overexpressing cells we put 500 L-K+ GFP+ cells/plate, exposed cells to various concentrations of G-CSF (0–10 ng/ml) and scored colonies at day 7.

In order to assess the sensitivity of cells with and without miR-125 OE we injected mice with one dose of G-CSF. On day 3 we isolated L-K+ GFP+ cells and placed them in CFU-GM assay, on day 7 we scored colonies.

### Isolation of L-K+ cells from different skeletal locations after transplantation

Transplanted recipients were sacrificed by cervical dislocation under isoflurane anesthesia. Bones were isolated separately into five groups: 1) bones of fore limbs and sternum (front); 2) left femur, tibia and left part of the pelvic bone (left hind bone); 3) right femur, tibia and right half of pelvic bone (right hind bones); 4) bones of spine, 5) bones of skull. After removal of muscles, cells were isolated by crushing in lysis solution (NH_4_Cl) and filtered through 100 -µm filter to remove debris. Nucleated cells were washed, counted and stained with an antibody cocktail. Cells were washed and resuspended in a 1 µg/ml solution of propidium iodide (PI). Antibodies were directed against c-Kit, a panel of lineage markers (Ter119, Gr1, B220, Mac1, CD3ε). Cells were sorted directly into tubes, spun down and used for further barcode analysis.

### Clonal analysis of L-K+ cells

Genomic DNA was extracted from L-K+ cells as described previously^14^ and barcoded DNA was amplified with primers against internal vector sequence^30^. For deep sequencing barcode region from each cell sample was amplified with specially tagged eGFP primers and un-tagged reverse primer (Rev YY1iPCR 5′CCAAACCTACAGGTGGGGTCTTTCATTC3′). DNA products were pooled together and sequenced as a single sample in Illumina 2500 HiSeq machine, rapid run, 100 bp long, according to the protocol of the manufacturer.

### Clonal analysis of blood samples

Clonal analysis in blood was performed as previously described^14^. In brief blood samples were collected every 4 weeks for 4–6 months and at the moment of sacrifice. Erythrocytes were lysed in NH_4_Cl buffer, and cells were stained with fluorophore-conjugated antibodies against Gr1, B220, CD3ε, Ter119^13^, washed and resuspended in PI solution. Mature blood cells were purified by FACS. Viable (PI-) granulocytes (Gr1+ side scatter high), T cells (CD3ε), B cells (B220+) and erythroid cells (Ter119+) were sorted. Barcode sequence was determined as described above.

### Barcode data processing

Data extraction was performed using in-house developed scripts in Python, Perl, R and VBA, as recently described^14^. Briefly high quality barcode reads were extracted using Motif Occurrence Detection Suite^31^. Barcodes were sorted by read frequencies from highest to lowest, and all minor barcodes frequencies different by a single nucleotide (one base distance) were merged with the dominant major barcode. Unique barcodes with frequencies under 10 were removed (technical threshold). All samples with <1,000 reads were excluded from further analysis.

### Statistical analysis

For testing the statistical significance of variation of clone size among different bone groups, we calculated the relative standard deviation (RSD). For comparing RSD values between groups we used non-parametric Mann-Whitney test that allows comparison of not-normally distributed data. P-values were calculated using Prism.

Correlation and linear regression analyses and p-values were derived using Prism (GraphPad Software), Python and R. Clones with potetnially multiple hits were identified on the basis of linear regression analysis, using a custom Python script and linregress () function from scipy stats library. The barcodes were considered as linked (multiple integration of the virus into the same cell-multiple hits) if the linear regression between any pair of BCs were in the range (1-slope) <0.1. For such thresholds, p-values were far below 10^−10^.

Barcodes were normalized, sorted from highest to lowest and used for the linear regression with Pearson correlation to count linked barcodes and therefore estimate the number of cells with single, double and higher vector copy number.

To visualize changes in the contribution and rank order of barcodes in serial transplantation experiments we included barcodes present >10 in the primary donor contributing >0.5% to Gr1+ cells and analyzed their contribution in secondary recipients. For data visualization we used custom Python script and R river plot algorithm.

In this paper we have mainly used correlation coefficients (Pearson) as a measure of similarity between samples. This function is quantitative and have particular limitations, nevertheless is well known and broadly used, and when properly used provides sufficient statistical information.

All methods were carried out in accordance with regulations at the University Medical Centre of Groningen.

All experimental protocols were approved by the Animal Care Committee at the University of Groningen and National Bureau for Genetically Modified Organisms (*Bureau GGO*).

## Supplementary information

Supplementary information

Supplementary dataset
