# Supplementary material for: MiR-125a enhances self-renewal, lifespan, and migration of murine hematopoietic stem and progenitor cell clones
Source: Sci Rep. 2019 Mar 18;9:4785. doi: 10.1038/s41598-019-38503-z (PMC6423273; doi:10.1038/s41598-019-38503-z)
Supplement: Supplementary file 1 — Supplementary information [file 41598_2019_38503_MOESM1_ESM.pdf]

MiR-125a enhances self-renewal, lifespan, and migration of murine hematopoietic stem and progenitor cell clones.

Edyta Ewa Wojtowicz[1*], Mathilde Johanna Cristina Broekhuis[1], Ellen Weersing[1], Alexander Dinitzen[1], Evgenia Verovskaya[1], Albertina Ausema[1], Martha Ritsema[1], Erik Zwart[1], Gerald de Haan[1*] and Leonid V. Bystrykh[1*]

[1]Laboratory of Ageing Biology and Stem Cells, European Research Institute for the Biology of Ageing, University Medical Centre Groningen, University of Groningen, Antonius Deusinglaan 1, 9700 AV Groningen, the Netherlands

*Correspondence: edyta.wojtowicz@ki.se (E.E.W), g.de.haan@umcg.nl (G.d.H.), l.bystrykh@umcg.nl (L.B.)

Running title: MiR-125a enhances clonal HSC self-renewal, lifespan, and migration.

Supplementary figures

Suppl. Figure 1. Gating strategy for FACS purification of LT-HSC or progenitors from donor mice

Our sorting strategy consisted of 2 consecutive purification steps. Firstly, we presorted Lin-Sca+1+cKit+ cells to enrich for L-S+K+ cells. In next step, we stringently purified LT-HSC and progenitor cells.

Suppl. Figure 2. The kinetics of contributing clones at 12 weeks post transplantation

The kinetics of clones contributing to (A) Gr1+ cells, (B) Ter119+, (C) B cells or (D) T cells. Each symbol indicates an individual mouse. The same symbol in vertical columns corresponds to the same mouse (clonal composition in different lineages).

Suppl. Figure 3. The kinetics of consistent clones at 12 weeks post transplantation

The kinetics of clones consistently detected in (A) Gr1+ cells, (B) Ter119+, (C) B cells or (D) T cells. Each symbol indicates an individual mouse. The same symbol in vertical columns corresponds to the same mouse (clonal composition in different lineages). Symbols between Suppl. Fig 2 and 3 are consistent.

Suppl. Figure 4. Contribution of different clones to mature blood lineages

A.) Pearson correlations between barcode composition of 4 cell types at 4 or 5 time points from mice transplanted with (A) control LT-HSC, (B) miR-125a overexpressing LT-HSC and (C) progenitors

Suppl. Figure 5 Serial transplantation 'activates' clones that were dormant in the primary donor

In primary recipient clones contributing to >0.5% Gr-1+ cells in the peripheral blood were ranked from highest to lowest (depicted as 'peripheral blood'). We show only those primary barcodes that were subsequently detected in secondary and tertiary

recipients. Next, clones detected in secondary recipients were ranked from highest to lowest contributors and traced back to their donor (a higher position in 2nd recipient reflects a higher contribution). Secondary and tertiary clones that were not identified in PB of the primary donor but that were found in primary bone marrow are depicted in white ('bone marrow' grey rectangles). The thickness of lines connecting the same clone in the primary and secondary or tertiary recipient (in case of miR-125a) reflects the level of contribution. Panels A and B show transplant series using CV LT-HSC, while panels C. and D. show data from miR-125a OE LT-HSCs

Suppl. **Figure 6. Serial transplantation of miR-125a OE LT-HSC**

Two additional examples of serial transplantation of cells OE miR-125a (for a more detailed description, please see legend of Fig. 3).

Suppl. Figure 7. Proliferative effects due to multiple integrations of the vector

A.-D.) Relationship between the expected (Poisson distribution) and observed number of single and multiple hit clones in mice transplanted with CV LT-HSC, miR-125a LT-HSC, miR-125a progenitors and CV progenitors. E.) The ranking position (based on the cumulative clone contribution to bone marrow and blood) of barcodes with single, double and higher vector copy number per cell in mice transplanted with control or miR-125a overexpressing LT-HSC or progenitors. F.) The ranking position of barcodes with multiple vector integrations in the serial transplantation based on the cumulative barcode contribution to the bone marrow and blood at 12-20 weeks post-secondary transplantation. G.) Observed number of single and multiple hit clones in mice serially transplanted with control (n=9) or miR-125a overexpressing LT-HSC (n=11).

Suppl. Figure 8. miR-125a overexpressing, but not control clones exert symmetrical distribution in the skeleton

A.) Skeletal distribution of barcoded clones in control LT-HSC, B.) miR-125a overexpressing LT-HSC or C.) progenitors 24 weeks post transplantation. D.) Summary of all clones identified in 1 mouse transplanted with control LT-HSC, 24 weeks post transplantation, E.) same as D but for miR-125a overexpressing LT-HSC,

F.) the same as D but for miR-125a overexpressing progenitors. G.) Summary of the RSDs for clones found in mice transplanted with CV LT-HSC (n=3), miR-125a LT-HSC (n=3), miR-125a progenitors (n=2) at 24 weeks post transplantation. Horizontal lines indicate the mean value. The difference between the groups (control HSC and miR-125a HSC or control LT-HSC and miR-125a progenitors) was assessed by two-tailed Mann-Whitney test (***, P< .0001).

Supplementary Table 1 Experimental details of Experiment 1 and 2.

Transduction efficiencies, number of transplanted GFP+ cells injected per recipient, total number of transplanted and analyzed animals in both experiments.

Supplementary Table 2 Evaluation of repopulating potential of Lineage- cells isolated from peripheral blood, spleen or bone marrow in CV or miR-125a OE cells

The table includes cell doses, source of cells and the chimerism level (expressed as the % of GFP+ Gr1+ cells in the donor derived fraction). Donor contribution constituted 40-90% of the total Gr-1+ population (based on CD45.1 and CD45.2 marker expression).

Supplementary files

Suppl. Power Point, BM saturation model, gif movie

Suppl. Excel file containing clone contribution to PB (relates to Fig. 1E).

Supplementary Figure 1

presort

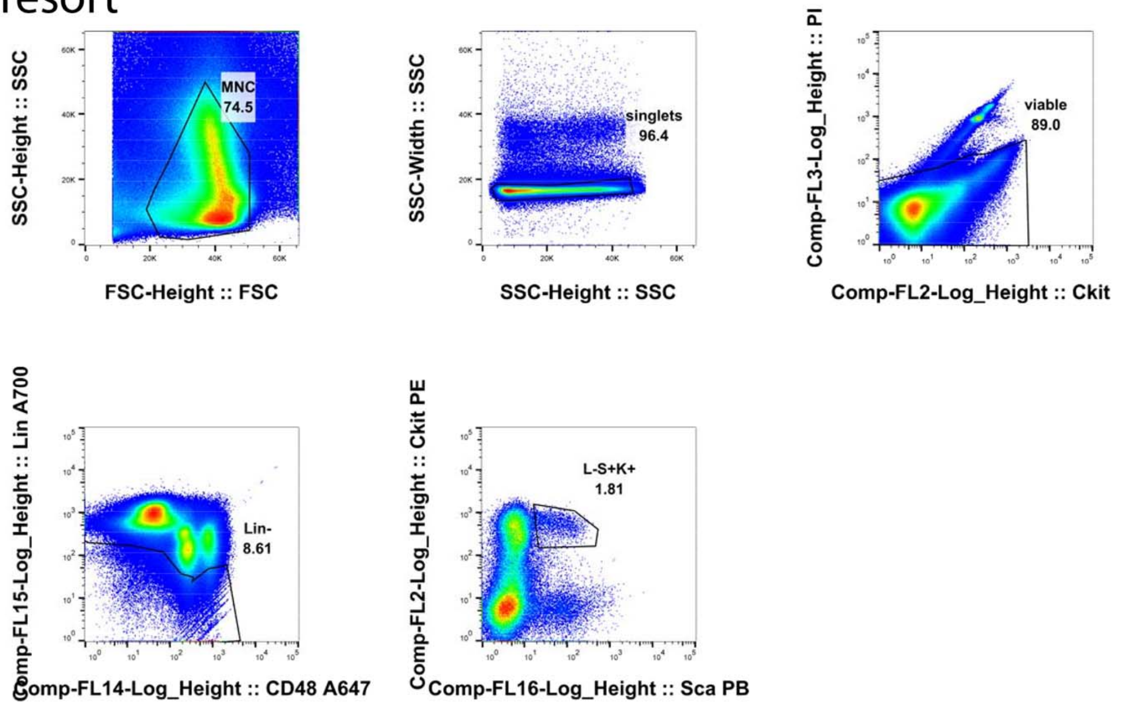

resort

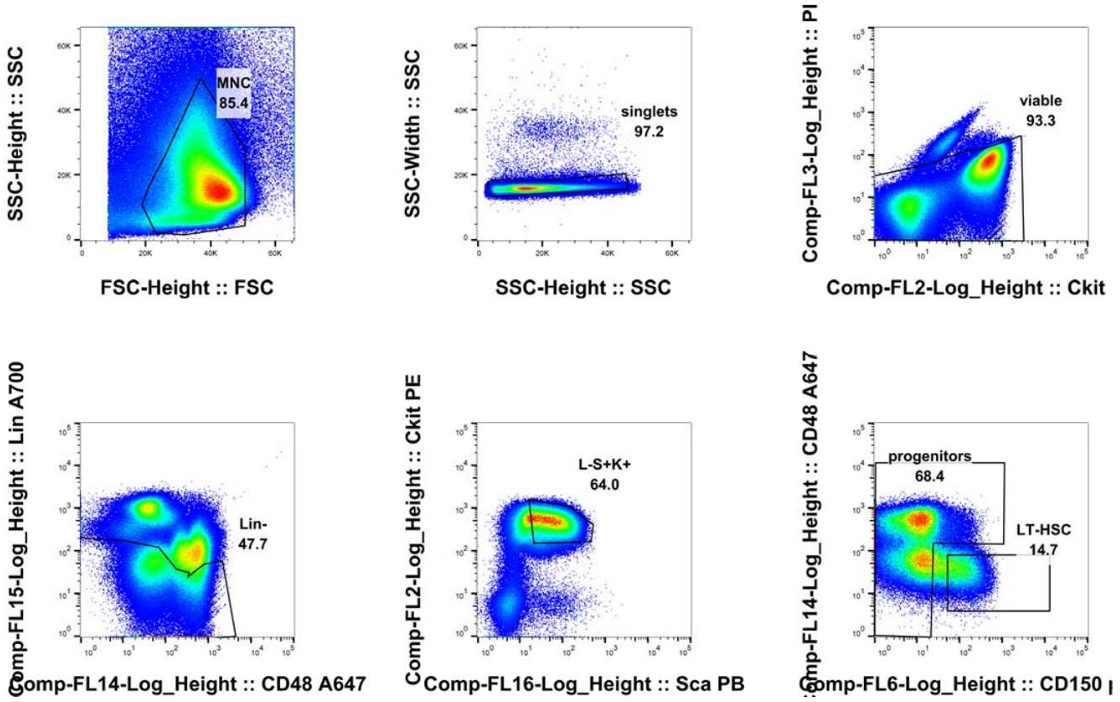

Supplementary Figure 2

Consistent clones

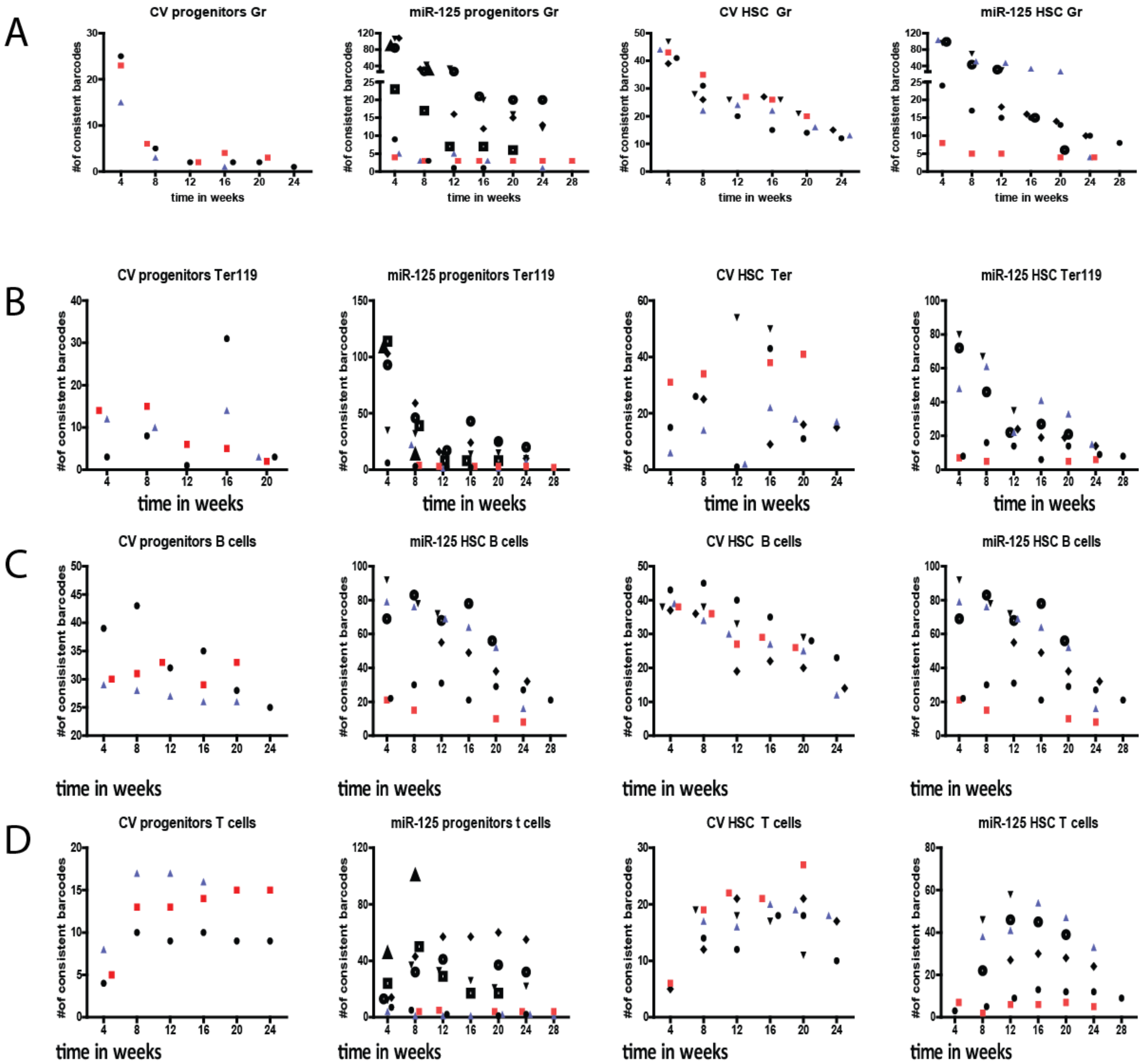

Supplementary Figure 3

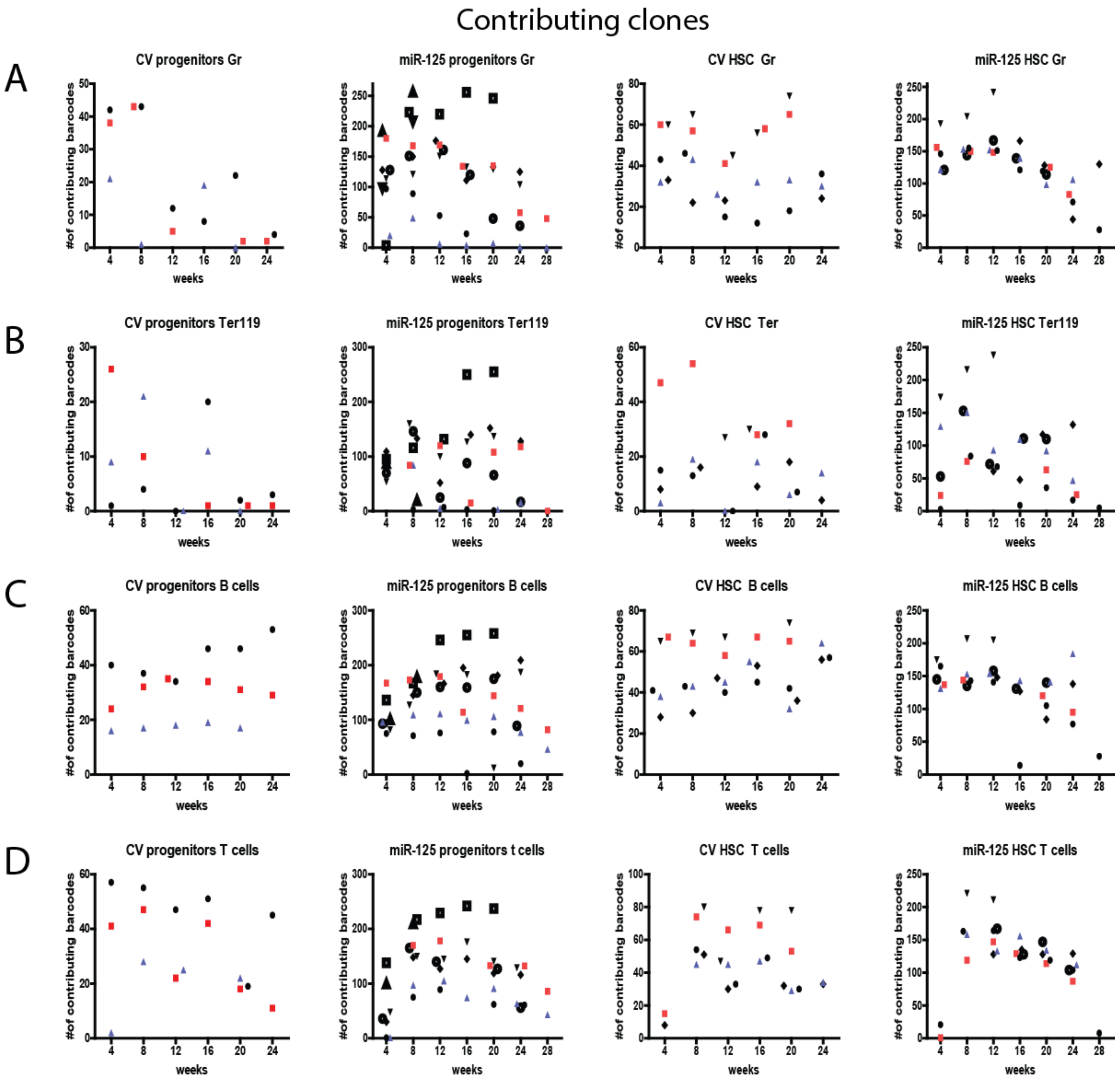

Supplementary Figure 4

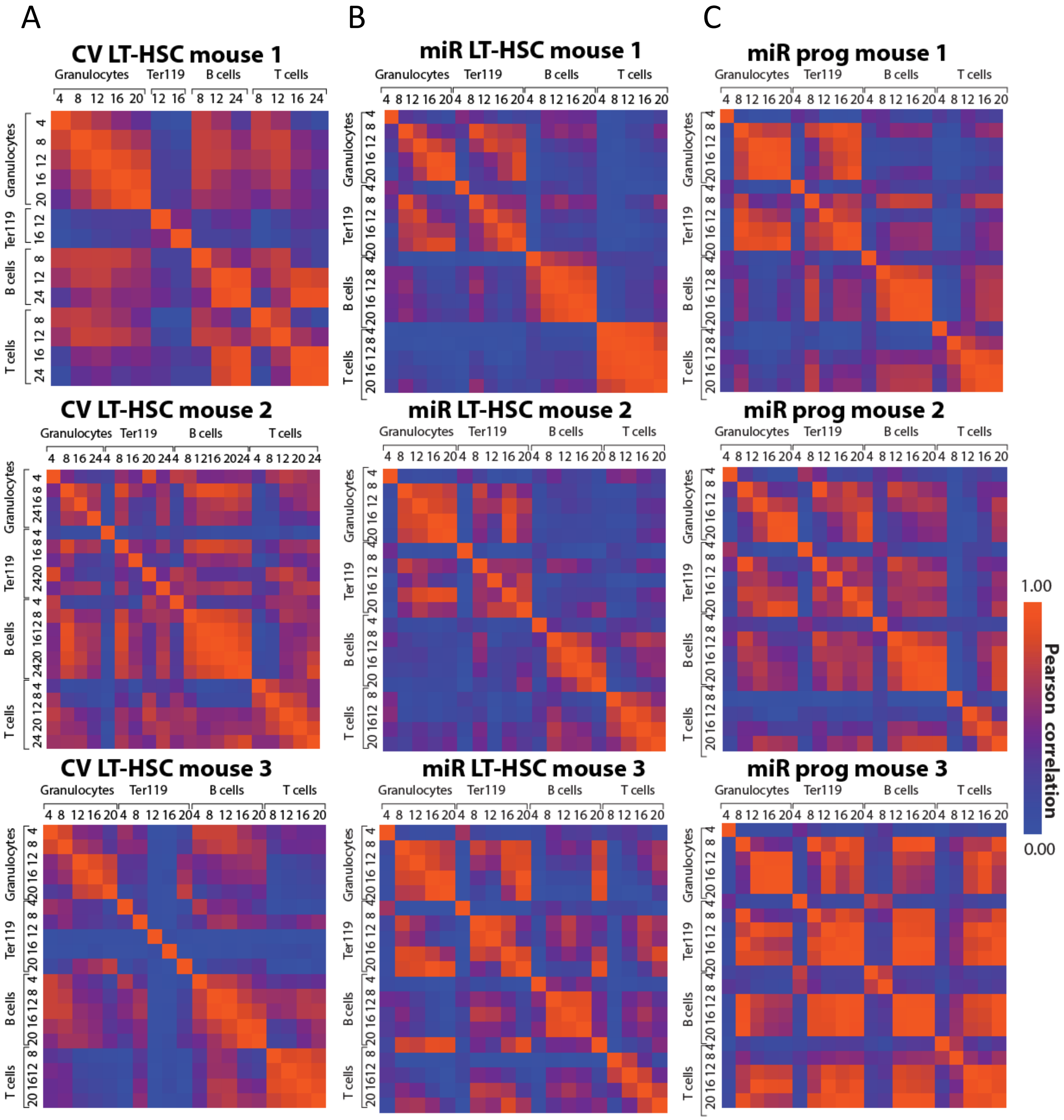

Supplementary Figure 5

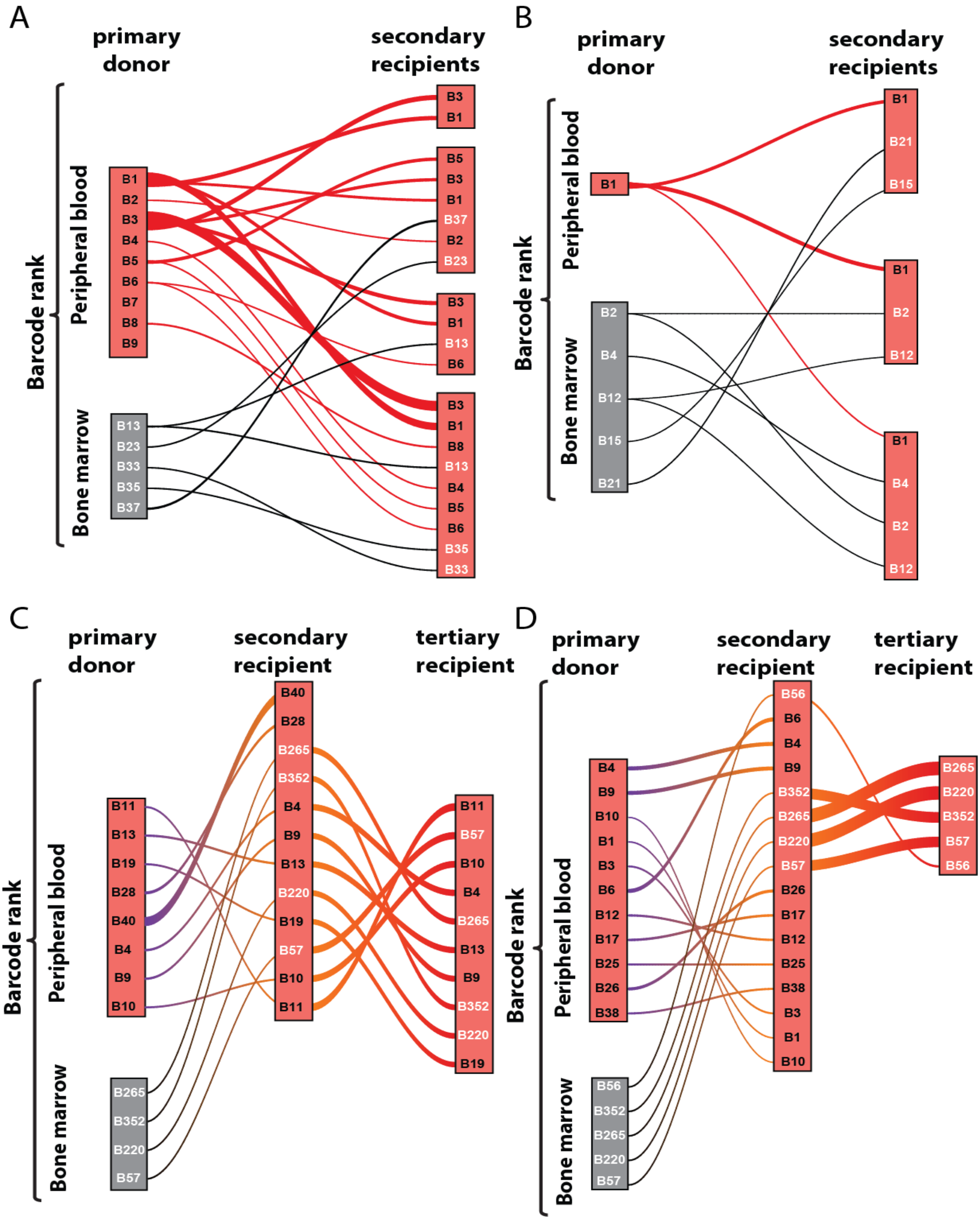

Supplementary Figure 6

A

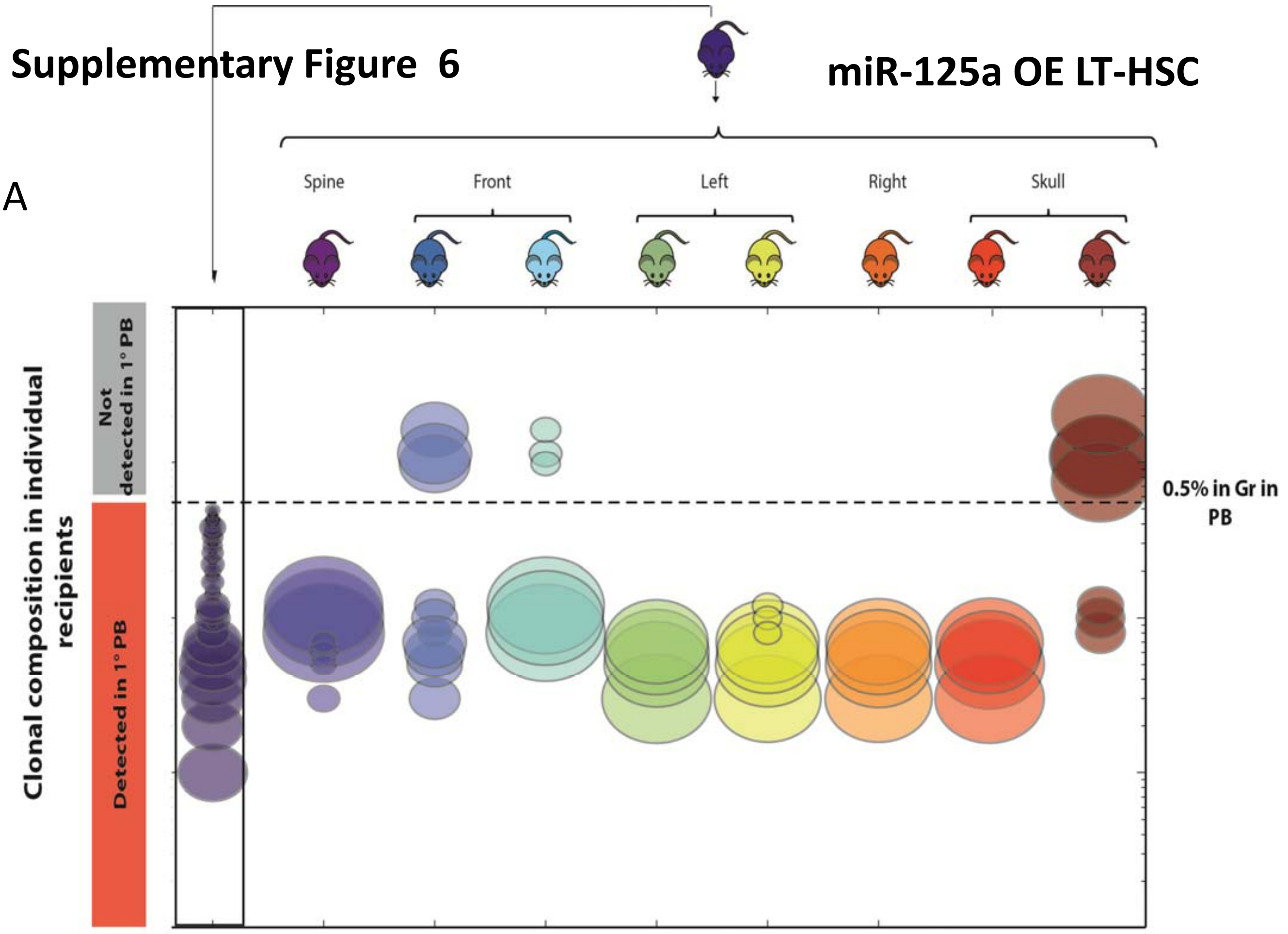

B

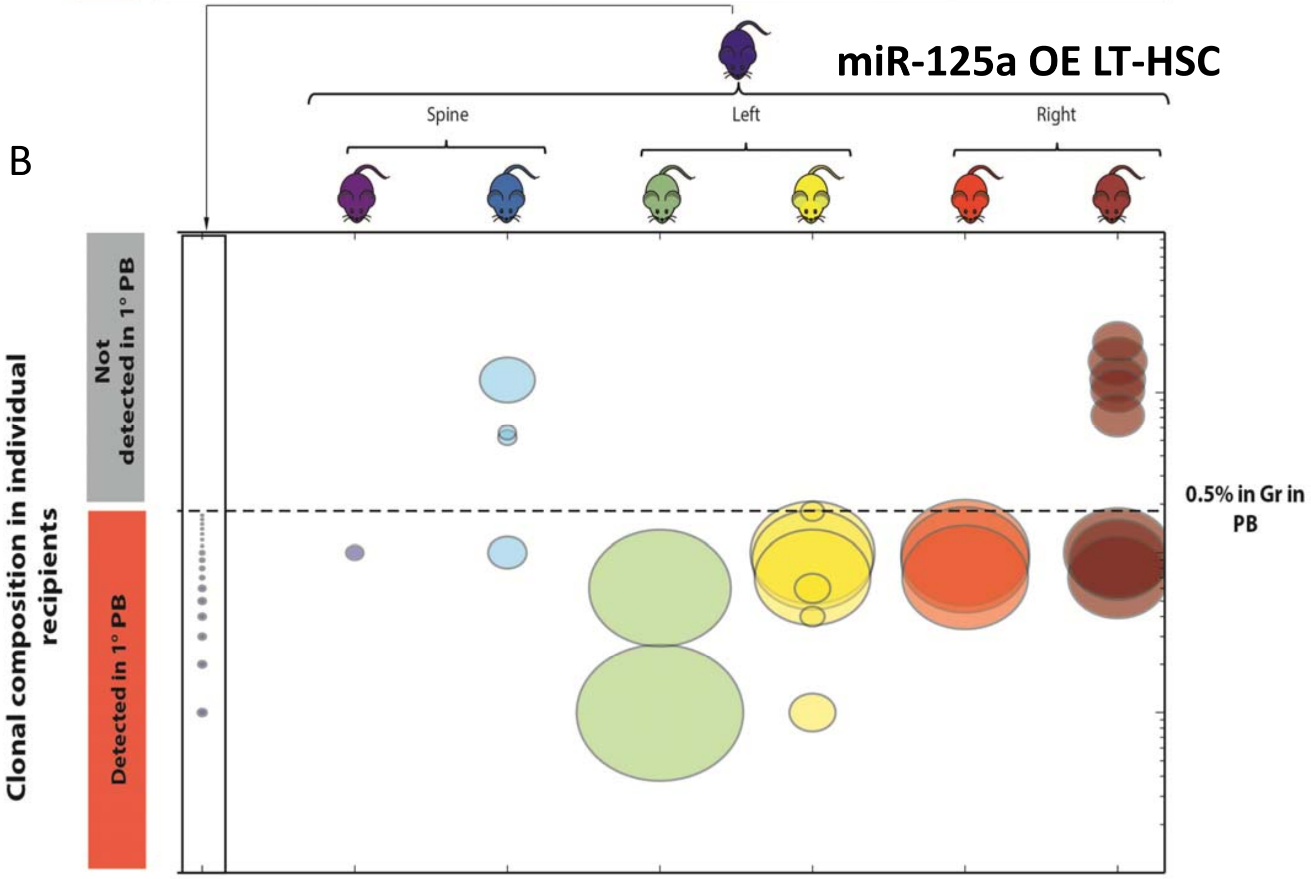

Supplementary Figure 7

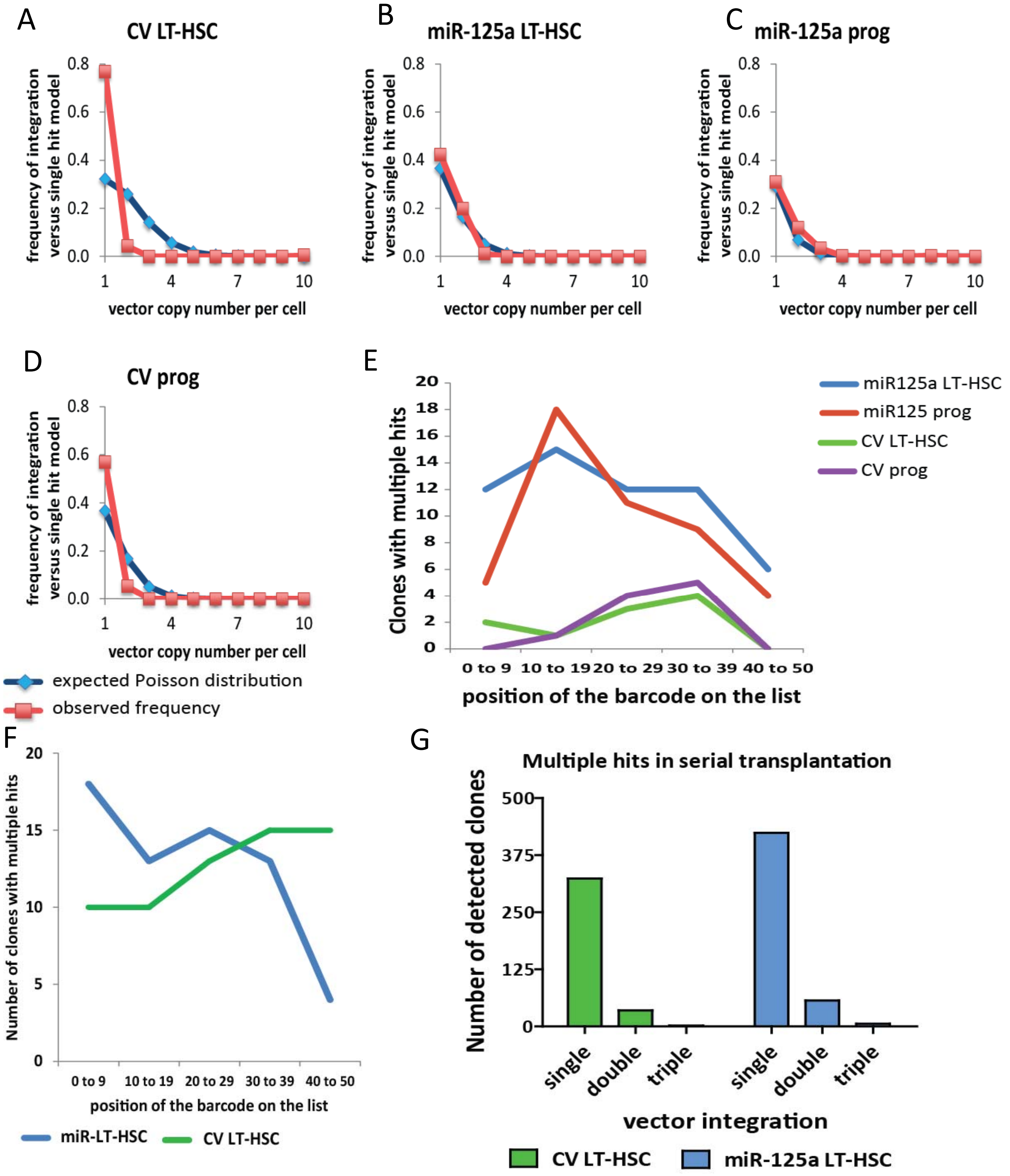

Supplementary Figure 8

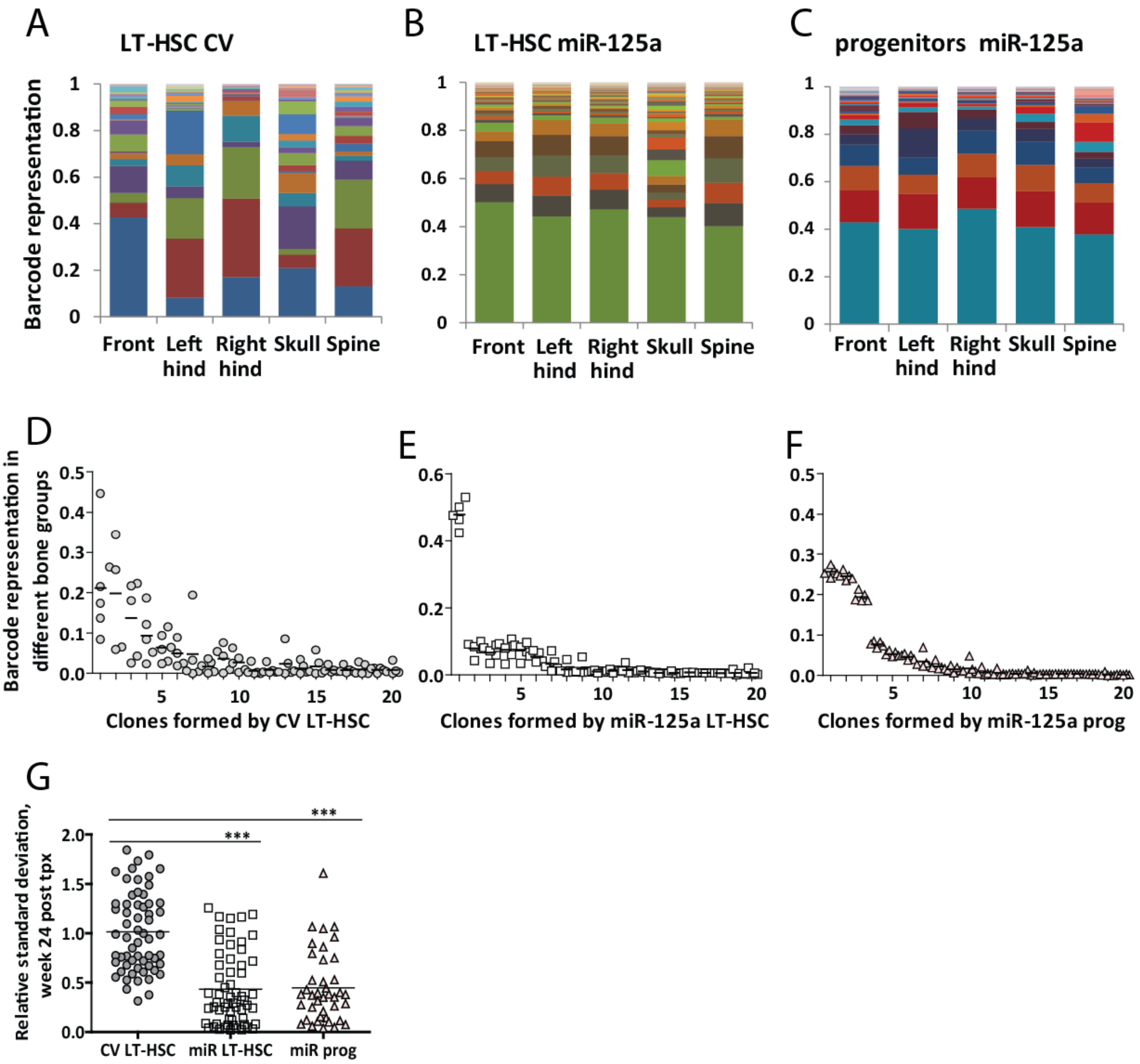

Supplementary Table 1

Experiment	Cell type	Full transduction efficiency in %	Number of GFP+ cells/mouse	Number of recipients
Exp. 1	125a prog	12,6	1180,7	2
	125a HSC	22,5	1881,4	2
Exp. 2	CV prog	60,0	39840,0	3
	CV LT-HSC	80,0	16213,3	5
	125a prog	37,0	15758,3	5
	125a HSC	51,1	4324,2	6

Supplementary Table 2

	mouse ID		cell source	cell dose	chimerism at 10 to 12 weeks post secondary transplantation in Gr1+ cells (% of GFP+ Gr1+ cells in donor derived fraction)
exp.1	mouse1	CV	PB	988	0
	mouse2	CV	PB	988	0
	mouse1	CV	SPL	146280	0
	mouse2	CV	SPL	146280	0
	mouse1	CV	BM	27736	63
	mouse2	CV	BM	27736	50
	mouse1	miR-125a	PB	72816	0
	mouse2	miR-125a	PB	72816	0
	mouse1	miR-125a	SPL	37732	87
	mouse2	miR-125a	SPL	37732	19
	mouse1	miR-125a	BM	171200	99
	mouse2	miR-125a	BM	171200	99
exp.2	mouse1	CV	PB	214502	0
	mouse2	CV	PB	214502	0
	mouse1	CV	SPL	750000	0
	mouse2	CV	SPL	750000	0
	mouse1	CV	BM	2515556	55
	mouse2	CV	BM	2515556	39
	mouse1	miR-125a	PB	171622	0
	mouse2	miR-125a	PB	171622	0
	mouse3	miR-125a	PB	80151	0
	mouse4	miR-125a	PB	80151	0*
	mouse1	miR-125a	SPL	750000	100
	mouse2	miR-125a	SPL	750000	100
	mouse3	miR-125a	SPL	750000	100
	mouse4	miR-125a	SPL	750000	100
	mouse1	miR-125a	BM	1586667	92
	mouse2	miR-125a	BM	1586667	76
	mouse3	miR-125a	BM	2177778	100
	mouse4	miR-125a	BM	2177778	100

On stem cells asymmetry

- There are several bone marrow (BM) compartments that can host/accommodate stem cells (HSC). In our experimental set up we have 5 such compartments (Front, Left hind, Right hind, Skull and Spine), where HSCs can end up after the transplantation.
- Usually in our experiments we transplant relatively low number of HSCs per recipient. Our transduction protocol allows only 1 cell division before the transplantation; therefore at max we can transplant 2 cells harbouring the same barcode/recipient. Thus some cells will miss certain locations (not enough cells to equally distribute them over 5 compartments). Therefore, at the beginning cells are asymmetrically distributed in the BM.
- During the first days up to weeks HSCs are forming clones through self-renewal divisions. If only one HSC would end up in a certain bone marrow compartment it would take considerably long time to 'colonize' the whole compartment.
- Cells can self-renew ergo expand only till certain, critical point when they saturate all available niches in the BM compartment. When they approach this point, they will start entering the circulation and 'look' for other niches in remaining BM compartments. If there was only one barcoded HSC, it will take considerably longer time to saturate all niches in the BM. However, more than one HSC has 'seeded' the BM compartment, the degree of individual expansion of each of these clones will be smaller and required for the BM saturation will be shorter. Overall we would expect much faster equilibration with bigger number of clones due to smaller local expansion, shorter time to saturate available niches, therefore we would observe enhanced reshuffling of clones between different BM compartments.
- Furthermore, the equilibration of cells would be slower if a significant group of cells would go into differentiation.
- Another option is an increased self-renewal. Eg if cells are dividing more frequent they will saturate available niches faster, so not only the number of clones but also the frequency of cell divisions (in case of HSC the self-renewal) would matter.

Model rationale

There will be always one empty bucket at least

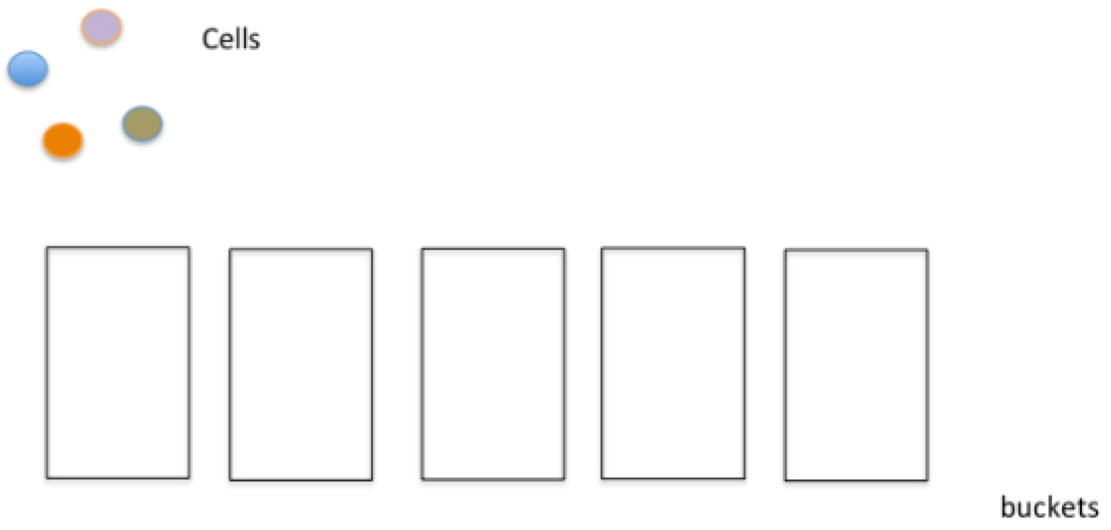

The model: script bone_asymmetry.py

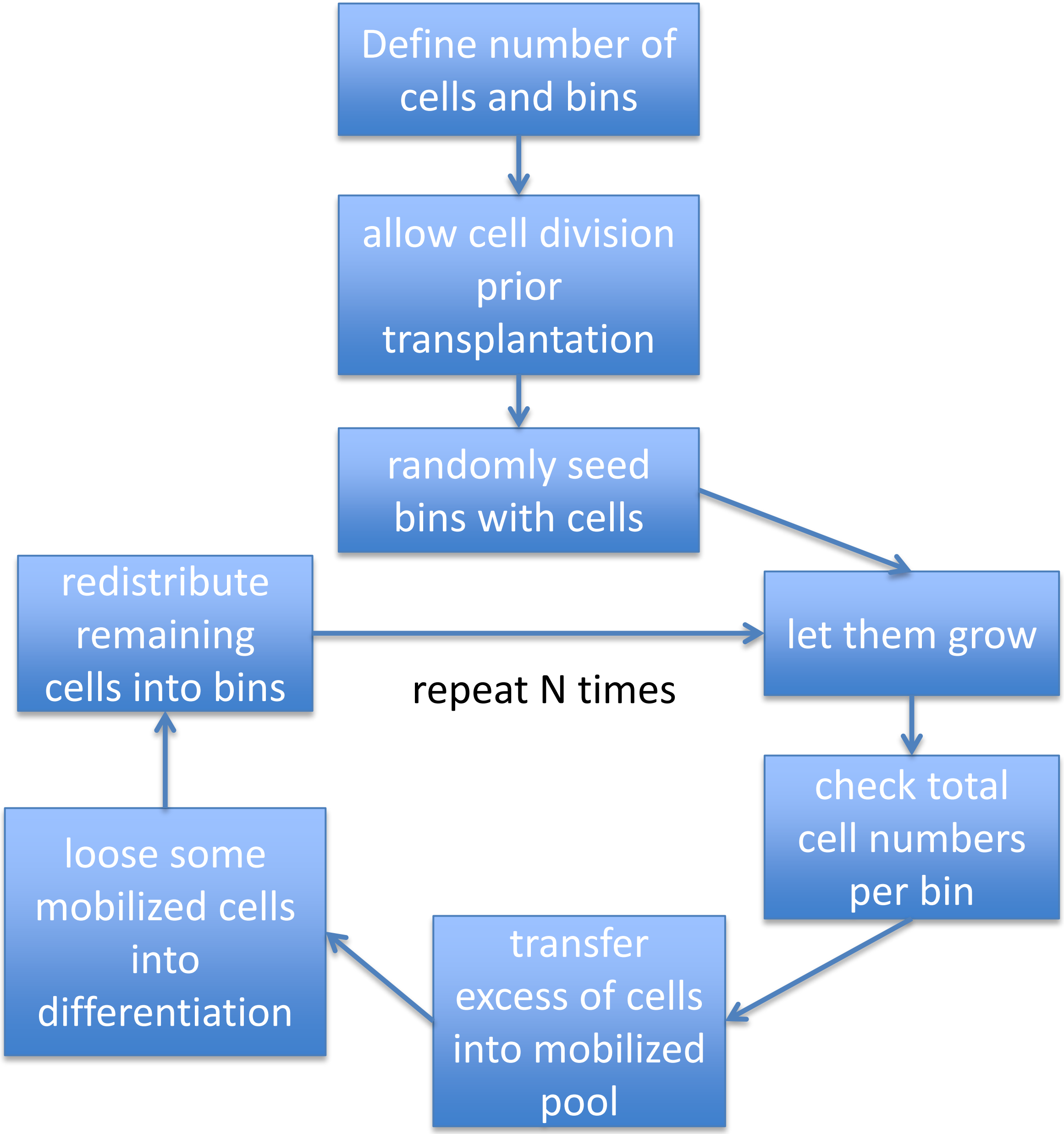

Plot stdev/average versus generations (== iterations of the model)

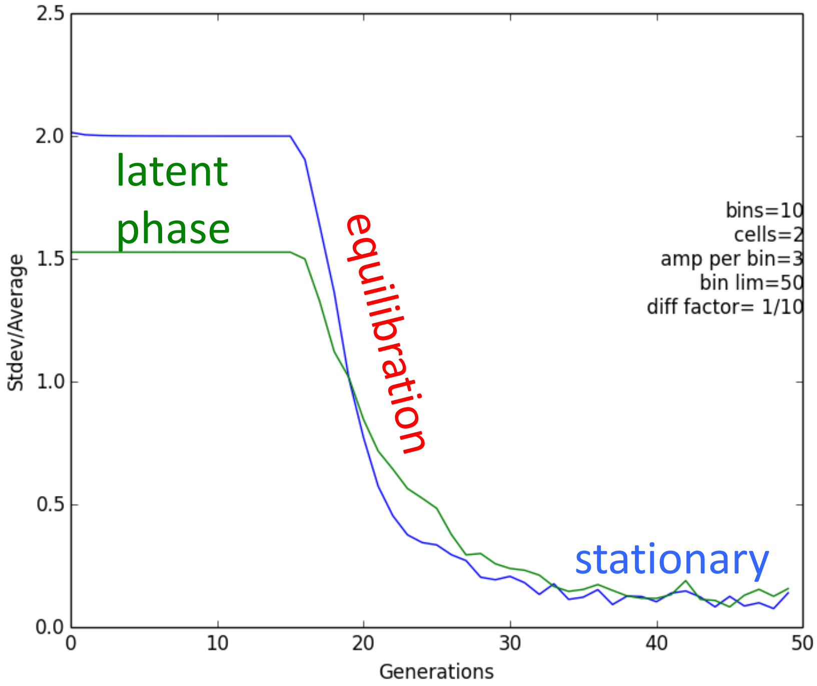

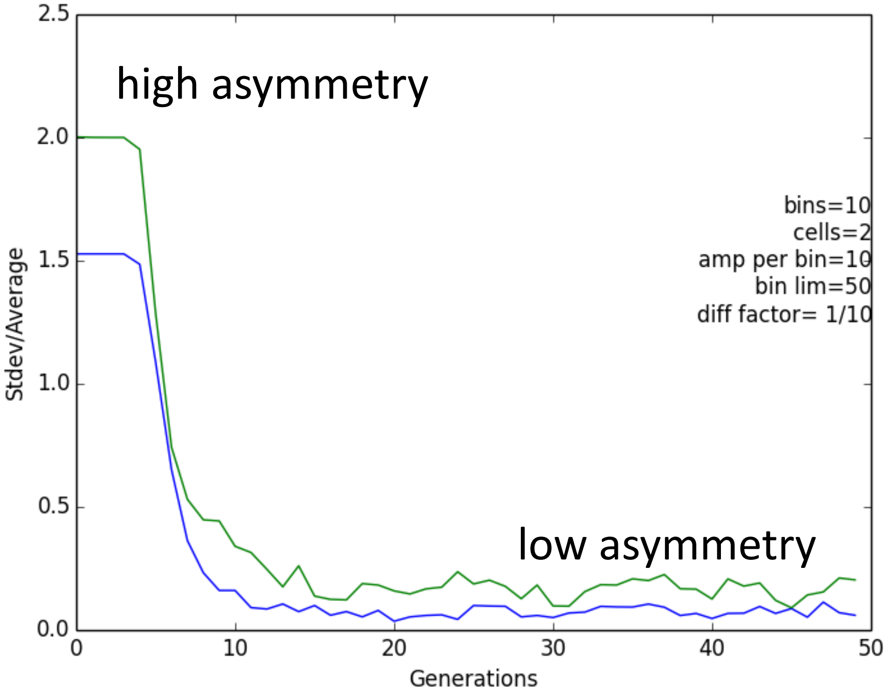

Length of the latent phase=

bin capacity

initial number of cells + cells amplification per generation * generati ons - differentiat ion

In latent phase

- the length is bigger with
  - less cells
  - less self-renewal
  - more cells are lost on differentiation

- and other way around

Length of the latent phase=

bin capacity

---

initial number of cells + cells amplification per generation * generations - differentiation

Tested model settings

Parameters\Movies	10_5	5_10	5_6_3_2	5_6_3_10	5_6_3_20	5_6_3_30	5_6_10_10	5_6_10_30	5_6_10_50
bones	10	5	5	5	5	5	5	5	5
clones	5	10	6	6	6	6	6	6	6
amp.factor per iteration	3	3	3	3	3	3	10	10	10
bone limit	50	50	50	50	50	50	50	50	50
differentiation factor 1/x	30	30	2	10	20	30	10	30	50

Notes: bone limit was constant through simulations

Example .gif movie file

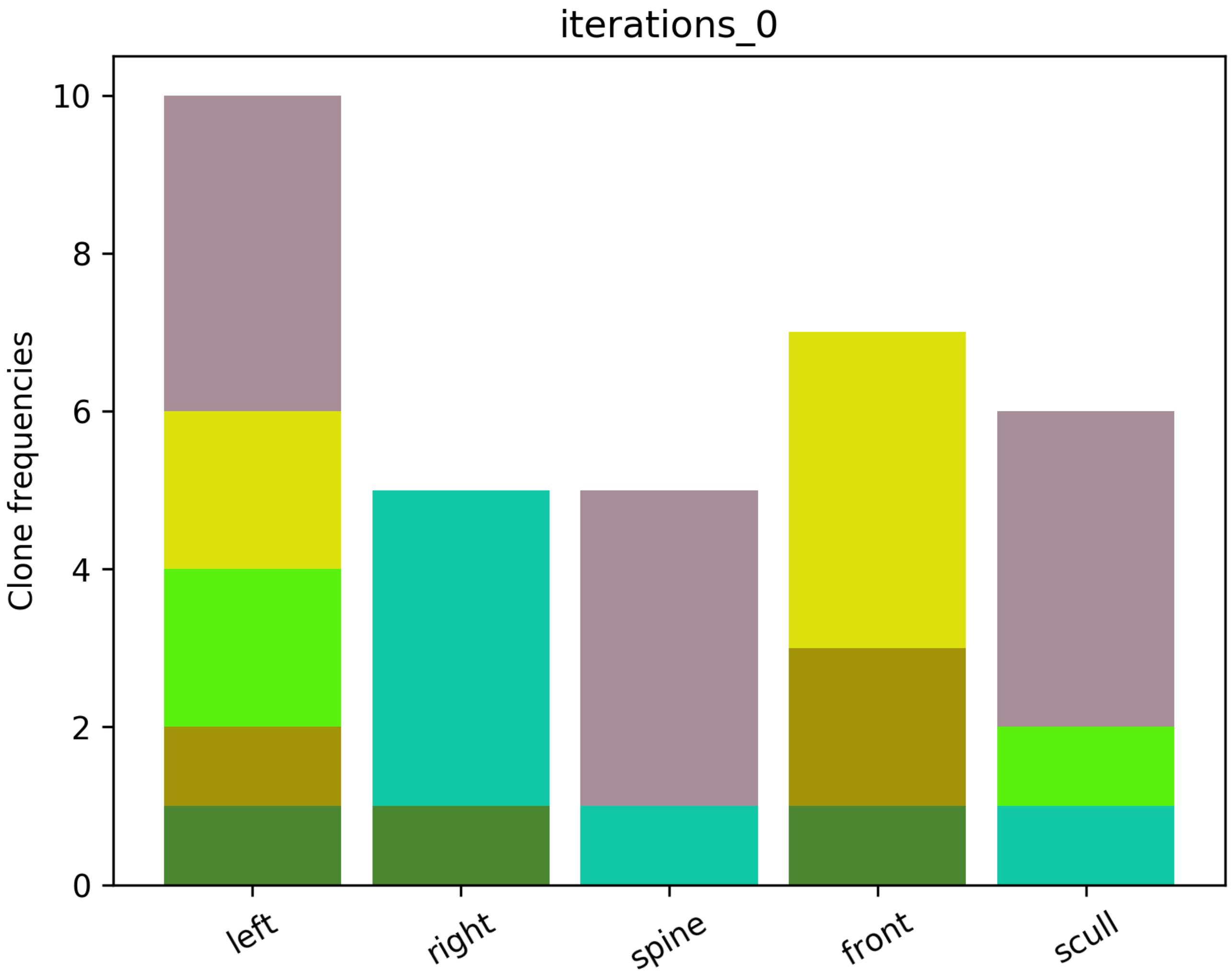

it should be active in the presentation mode
